# Novel PPAR-γ Agonist from the Soft Coral *Sarcophyton crassocaule*: Modulating Glucose Uptake and Lipid Droplet Formation

**DOI:** 10.3390/md23120450

**Published:** 2025-11-24

**Authors:** Jian-Ang Zeng, Min Sun, Yi Qi, Song-Wei Li, Li-Ting Zhang, Si-Min Pan, Yue-Wei Guo, Ming-Zhi Su, Hui Luo

**Affiliations:** 1 Guangdong Engineering Technology Research Center for the Development and Utilization of Mangrove Wetland Medicinal Resources, The Key Lab of Zhanjiang for R&D Marine Microbial Resources in the Beibu Gulf Rim, School of Ocean and Tropical Medicine, Guangdong Medical University, Zhanjiang 524023, China; zjianang05@163.com (J.-A.Z.); qiyi7272@gdmu.edu.cn (Y.Q.); 2 Shandong Laboratory of Yantai Drug Discovery, Bohai Rim Advanced Research Institute for Drug Discovery, Yantai 264117, China; sunmin7974@163.com (M.S.); zlt142301@163.com (L.-T.Z.); pansimin0928@163.com (S.-M.P.); 3 National Engineering Research Center for Modernization of Traditional Chinese Medicine, Guangzhou 510632, China; 4 School of Medicine, Shanghai University, Shanghai 200444, China; songweili@shu.edu.cn

**Keywords:** soft coral, *Sarcophyton crassocaule*, cembrane-type diterpenes, X-ray diffraction, PPAR-γ agonist, glucose uptake, lipid metabolism

## Abstract

Two previously undescribed highly oxygenated cembrane-type diterpenes, namely sarcocraol A (**1**) and sarcocraol B (**2**), along with five known compounds (**3**–**7**), have been isolated from the soft coral *Sarcophyton crassocaule* collected off Ximao Island in the South China Sea. Their structures were determined through comprehensive spectroscopic analysis, QM-NMR calculations, TDDFT-ECD computation, X-ray diffraction analysis, and by comparison with literature data. Plausible biosynthetic pathways for these compounds were also proposed. All compounds were evaluated for peroxisome proliferator-activated receptors (PPARs) transcriptional activity using luciferase assay. The bioassay results demonstrated that compound **1** exhibits selective PPAR-γ agonistic activity. Furthermore, it promoted glucose uptake in HepG2 cells by 1.18-, 1.45-, and 1.90-fold at concentrations of 2.5, 5, and 10 μM, respectively, whereas rosiglitazone (10 μM) produced a 2.47-fold increase over the induced control. Compound **1** at 10 μM induced mild lipid accumulation in 3T3-L1 cells, showing a 1.63-fold increase relative to the control, which was much lower than the 3.28-fold increase observed in rosiglitazone (10 μM) group indicating its potential antidiabetic properties. These findings suggested that compound **1** could be a promising lead for the development of antidiabetic agents.

## 1. Introduction

Peroxisome proliferator-activated receptors (PPARs) are members of the nuclear receptor superfamily and can be activated by dietary fatty acids (FAs) and their metabolic derivatives [1,2]. PPARs are ligand-dependent transcription factors that consist of three subtypes: PPAR-α, PPAR-β/δ, and PPAR-γ [3]. These receptors play a crucial role in regulating the transcription of target genes and are deeply involved in modulating pathophysiological processes, including glucose homeostasis, lipid metabolism, energy balance regulation, inflammation, and fibrosis [4,5]. PPAR-γ is predominantly expressed in adipose tissue, where it accelerates fatty acid uptake into adipocytes and increases insulin sensitivity, thereby reducing fatty acid flow to the liver [6,7]. Activation of PPAR-γ enhances glucose uptake and glycogen storage, while concurrently promoting lipid absorption and lipogenesis in adipocytes, thereby collectively reducing blood lipid levels [8,9]. PPAR-γ agonists, particularly those of the thiazolidinedione class (TZDs; e.g., rosiglitazone, troglitazone, and pioglitazone), exert antidiabetic effects by regulating adipocyte differentiation and glucose metabolism [10]. However, the clinical application of TZDs is often limited by adverse effects such as weight gain, bone density loss, congestive heart failure, and fluid retention. Therefore, the discovery of novel PPAR-γ agonists with improved safety profile is crucial in the development of therapeutics for type 2 diabetes mellitus.

**Figure 1:**
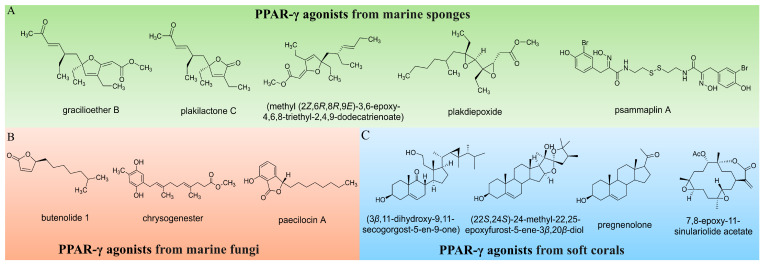
Representative marine-derived compounds with PPAR-γ agonistic activity. (**A**) PPAR-γ agonists from marine sponges. (**B**) PPAR-γ agonists from marine fungi. (**C**) PPAR-γ agonists from soft corals.

Natural products derived from marine organisms have attracted growing interest in drug discovery due to the remarkable diversity of their chemical scaffolds. In a recent review, Enrico D’Aniello et al. summarized numerous compounds targeting PPARs from marine sponges, fungi, and algae (Figure 1A,B) [11]. There are also a number of studies that have documented steroids derived from soft corals with PPAR-γ agonistic activity (Figure 1C) [12,13,14]. Thence, marine natural products serve as a promising reservoir of PPAR-γ agonists that urgently require further development.

Our research group has long been engaged in the chemical and biological investigation of marine natural products. In a previous study, we isolated 7,8-epoxy-11-sinulariolide acetate from the soft coral *Sinularia siaesensis* (Figure 1C). The compound enhanced PPAR-γ expression and represents the first marine cembranoid to be identified as a potential PPAR-γ activator [14]. Cembranoids, characterized by a 14-membered carbocyclic skeleton, are one of the primary metabolites found in soft corals belonging to the genera *Sinularia*, *Lobophytum* and *Sarcophyton*. This class of diterpenes are structurally diverse and frequently highly oxygenated, which contributes to their wide range of biological activities. Inspired by these findings, we developed a strong interest in the cembranoids derived from soft corals. Hence, we collected a specimen of the soft coral *S. crassocaule* from the South China Sea and conducted a systematic chemical investigation, leading to the isolation of two new highly oxygenated cembrane-type diterpenes, along with five known analogues. This paper describes the isolation, structural elucidation, proposed biosynthetic pathways, and biological evaluation of these compounds for PPARs agonist activity, as well as the effects of compound **1** on glucose uptake and lipid droplet formation [15,16,17].

## 2. Results and Discussion

### 2.1. Isolation and Structural Elucidation

**Figure 2:**
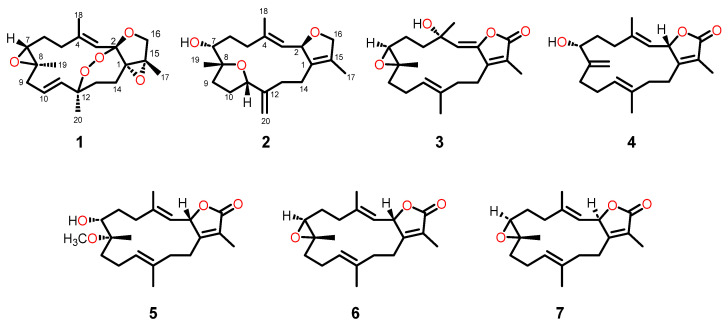
Chemical structures of compounds **1**–**7**.

Freshly samples of *S. crassocaule* were frozen immediately at −20 °C after collection and stored at that temperature before they were exhaustively extracted by acetone. The Et_2_O-soluble portion of the acetone extract of the title animal was subjected to repeated chromatography, including with silica gel, Sephadex LH-20, and RP-HPLC, to yield seven compounds (**1**–**7**) (Figure 2). The known compounds **3**–**7** were readily identified as metabolite 8 (assigned herein as **3**) in the reference paper [18], 7*α*-hydroxy-Δ^8(19)^-deepoxysarcophine (**4**) [18], 7*α*-hydroxyl-8*α*-methoxydeepoxysarcophine (**5**) [19], sarcophine (**6**) [20], and 2-*epi*-sarcophine (**7**) [20], respectively, by direct comparison of their NMR spectroscopic data with those reported in the literature.

**Figure 3:**
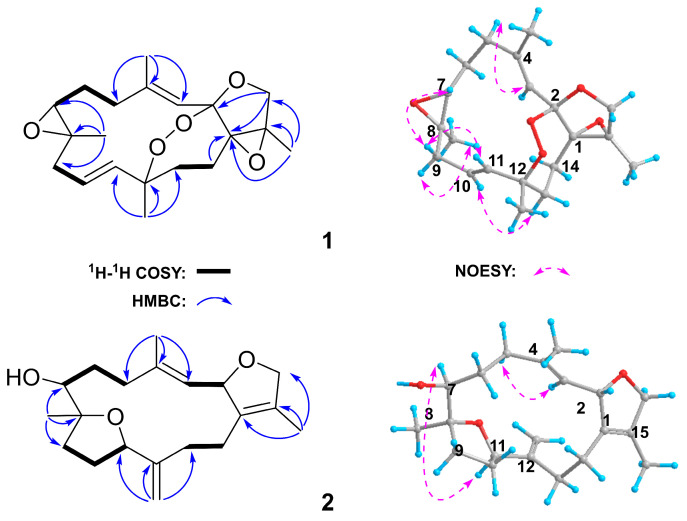
^1^H–^1^H COSY (bold line), key HMBC (blue solid arrow), and NOESY (red dashed arrow) correlations of **1** and **2**.

Compound **1** was isolated as a colorless crystal. The HR-ESIMS spectrum exhibited a peak at *m*/*z* 349.2004 ([M + H]^+^, calcd for 349.2009) establishing its molecular formula as C_20_H_28_O_5_, implying seven degrees of unsaturation. Detailed analysis of the 1D and 2D NMR spectra revealed the presence of one trisubstituted double bond [*δ*_H_ 5.37 (1H, s), *δ*_C_ 117.80 (CH, C-3) and *δ*_C_ 143.57 (qC, C-4)], one disubstituted double bond [*δ*_H_ 5.50 (1H, m), *δ*_H_ 5.64 (1H, d, *J* = 16.80 Hz), *δ*_C_ 123.43 (CH, C-10) and *δ*_C_ 135.97 (CH, C-11)], two epoxy moieties [*δ*_H_ 2.64 (1H, m), *δ*_C_ 61.58 (qC, C-7), *δ*_C_ 61.83 (CH, C-8); *δ*_C_ 65.72 (qC, C-1), *δ*_C_ 68.75 (qC, C-15)], and one oxymethylene group [*δ*_C_ 71.05 (CH_2_, C-17), *δ*_H_ 3.97 (1H, d, *J* = 9.81 Hz) and *δ*_H_ 4.07 (1H, d, *J* = 9.81 Hz)]. ^1^H–^1^H COSY correlations established the connectivity of H-5 (*δ*_H_ 2.34)/Ha-6 (*δ*_H_ 2.05), Hb-6 (*δ*_H_ 1.73)/H-7 (*δ*_H_ 2.64), H-9 (*δ*_H_ 1.76)/H-10 (*δ*_H_ 5.50)/H-11 (*δ*_H_ 5.64), and H-13 (*δ*_H_ 2.16)/H-14 (*δ*_H_ 1.74), while key HMBC correlations from CH_3_-18 to C-3/C-4/C-5, from CH_3_-19 to C-7/C-8/C-9, and from CH_3_-20 to C-11/C-12/C-13 enabled the construction of the 14-membered ring skeleton. Furthermore, HMBC correlations from CH_3_-17 to C-15, C-1, and C-16, and from H-16 to C-2, C-15, and C-1 indicated the formation of an oxygen-containing bridge between C-2 and C-16. These structural elements account for six degrees of unsaturation. The observed downfield chemical shifts in C-2 (*δ*_C_ 112.39) and C-12 (*δ*_C_ 86.53), along with the remaining degree of unsaturation, led us to postulate the presence of a peroxide bridge linking these two carbon atoms, as illustrated in Figure 3.

**Figure 4:**
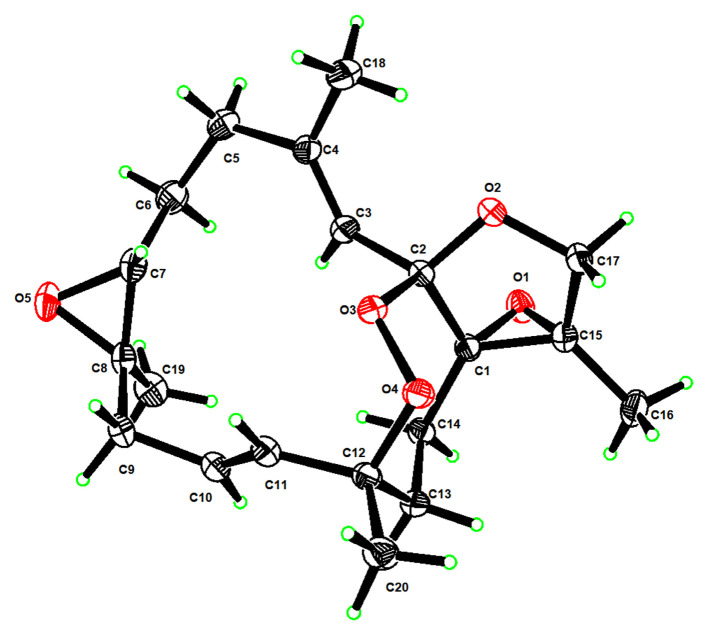
ORTEP drawing of the X-ray structure of **1** (the displacement ellipsoids are drawn at the 50% probability level).

In the NOESY spectrum, correlations between H-3 (*δ*_H_ 5.37) and Ha-5 (*δ*_H_ 2.34), between H-10 (*δ*_H_ 5.50) and Ha-13 (*δ*_H_ 2.16), as well as between H-11 (*δ*_H_ 5.64) and H-9 (*δ*_H_ 1.76) indicated that the Δ^3,4^ and Δ^10,11^ double bonds are both “*E*” geometry. Furthermore, the *E* configuration of the Δ^10,11^ double bond was supported by the coupling constant of H-11 (*J* = 16.08 Hz) as indicated in Table 1. The key NOESY correlations of H-7 (*δ*_H_ 2.64)/Ha-9 (*δ*_H_ 1.76) and CH_3_-19 (*δ*_H_ 1.41)/Hb-9 (*δ*_H_ 2.77) suggested the assignment of the relative configuration as 7R*, 8R*. In addition, suitable crystals of compound **1** were obtained in MeOH, and the X-ray crystallographic analysis using Cu K*α* radiation (λ = 1.54178 Å) firmly disclosed the planar structure of **1** and determined its absolute configuration to be 1*S*, 2*R*, 7*R*, 8*R*, 12*S*, 15*S* with a Flack parameter of −0.08(10) (Figure 4, CCDC 2428505). The compound **1** was named as sarcocraol A.

Compound **2** was obtained as a colorless oil with the molecular formula C_20_H_30_O_3_, as deduced from the HR-ESIMS ion peak at *m*/*z* 319.2265 ([M + H]^+^, calcd 319.2268), implying six degrees of unsaturation. The 1D and 2D NMR data revealed a tetrasubstituted double bond [*δ*_C_ 133.24 (qC, C-1), 128.5 (qC, C-15)], a trisubstituted double bond [*δ*_H_ 5.20 (1H, d, *J* = 9.62 Hz), *δ*_C_ 126.80 (CH, C-3), 139.56 (qC, C-4)], and a terminal double bond [*δ*_H_ 4.96 (1H, s), 5.00 (1H, s), *δ*_C_ 147.70 (qC, C-12), 115.00 (CH_2_, C-20)], along with three oxygenated methines (*δ*_C_ 67.85, 84.52, and 85.35), one oxygenated methylene (*δ*_C_ 78.51), and one oxygenated quaternary carbon (*δ*_C_ 80.74). These features accounted for three degrees of unsaturation, suggesting a tricyclic framework of compound **2**.

The ^1^H–^1^H COSY correlations of H-2 (*δ*_H_ 5.45)/H-3 (*δ*_H_ 5.20), H-5 (*δ*_H_ 2.28)/H-6 (*δ*_H_ 1.33)/H-7 (*δ*_H_ 4.34), H-9 (*δ*_H_ 1.59)/H-10 (*δ*_H_ 2.10)/H-11 (*δ*_H_ 4.63), and H-13 (*δ*_H_ 1.95)/H-14 (*δ*_H_ 2.20), together with the HMBC correlations from CH_3_-18 to C-3, C-4, and C-5; from CH_3_-19 to C-7, C-8, and C-9; from CH_2_-20 to C-11, C-12, and C-13, and from CH_3_-17 to C-15, C-1, and C-16, constructed a 14-membered ring cembrane-type skeleton. The remaining two degrees of unsaturation were attributed to oxygen bridges formed between C-8 (*δ*_C_ 80.74) and C-11 (*δ*_C_ 84.52), and between C-2 (*δ*_C_ 85.35) and C-16 (*δ*_C_ 78.51). Thus, the tricyclic planar structure of **2** was established (Figure 3).

**Figure 5:**
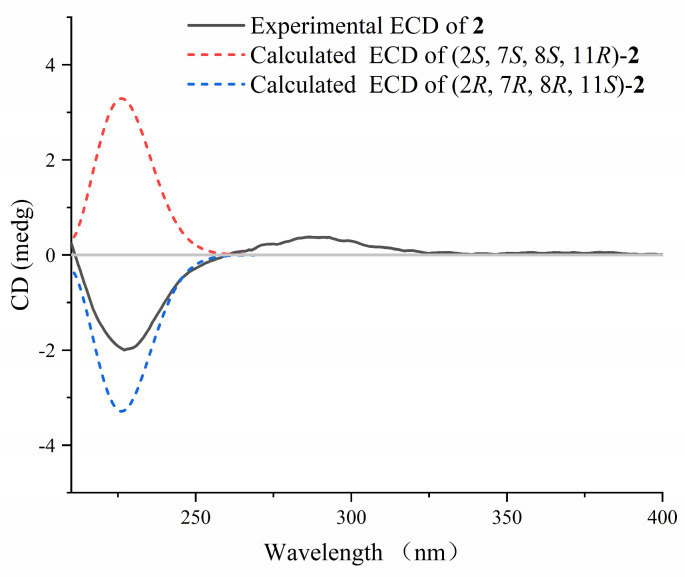
Experimental spectrum of **2** (black solid line), calculated ECD spectra of (2*S*, 7*S*, 8*S*, 11*R*)-**2** (red dashed line), and (2*R*, 7*R*, 8*R*, 11*S*)-**2** (blue dashed line).

In the NOESY spectrum, the correlation between H-3 (*δ*_H_ 5.20) and H-5 (*δ*_H_ 2.28) indicated an “*E*” geometry for the Δ^3,4^ double bond. In addition, the NOE correlation between H-7 (*δ*_H_ 4.34) and H-11 (*δ*_H_ 4.63) suggested that these two protons are located on the same side. The stereochemistry of these two atoms was tentatively assigned as 7S* and 11R*. Subsequently, QM-NMR calculations were performed on four possible stereoisomers including **2a** (2R*, 7S*, 8R*, 11R*), **2b** (2S*, 7S*, 8S*, 11R*), **2c** (2S*, 7S*, 8R*, 11R*), and **2d** (2R*, 7S*, 8S*, 11R*). The results revealed that the experimental NMR data of **2** exhibited the highest consistency (99%) with the calculated data of **2b**, and the relative configuration of **2** was elucidated as 2S*, 7S*, 8S*, 11R* [21,22,23]. The absolute configuration of **2** was determined by the application of TDDFT-ECD (time-dependent density functional theory/electronic circular dichroism) calculations at the B3LYP/6-311G(d,p) level [24,25]. As illustrated in Figure 5, the calculated ECD spectrum for the (2*S*, 7*S*, 8*S*, 11*R*)-enantiomer was opposite to the experimental data, whereas the spectrum for the (2*R*, 7*R*, 8*R*, 11*S*)-enantiomer provided an excellent match. Therefore, the absolute configuration of **2** was assigned as (2*R*, 7*R*, 8*R*, 11*S*) and named as sarcocraol B.

### 2.2. Plausible Biosynthetic Pathway of the Isolated Compounds

**Scheme 1:**
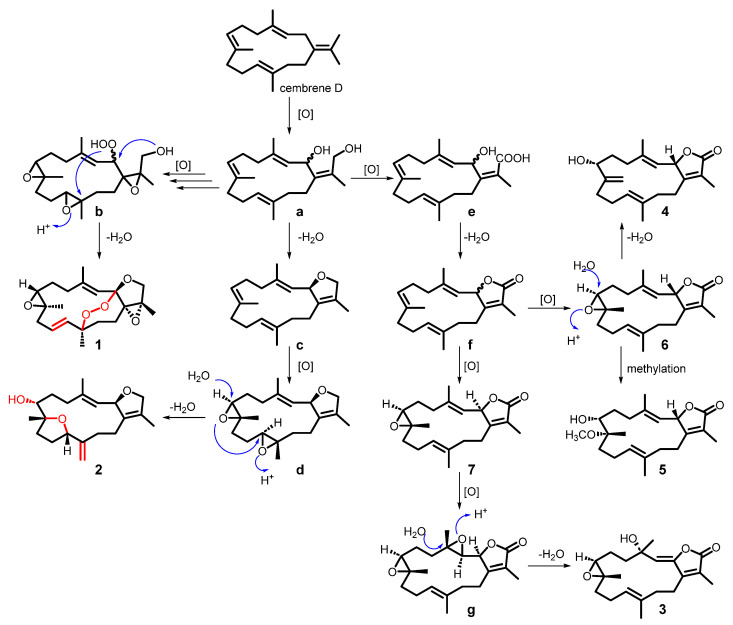
Plausible biosynthetic pathway of compounds **1**–**7**.

The close structural similarity among compounds **1**–**7** strongly suggested a common biogenetic origin. The proposed biosynthetic pathway originated from a simple precursor cembrene D. Initially, cembrene D underwent oxidation at different positions to yield intermediates **a**, **b**, and **e**, respectively. Intermediate **a** then underwent dehydration to yield intermediate **c**, characterized by a five-membered oxacyclic ring. Subsequent oxidation of **c** furnished intermediate **d**, which bears two epoxide functionalities at the C-7/C-8 and C-11/C-12 positions. The C-7/C-8 epoxide in **d** underwent ring opening via intramolecular nucleophilic attack by the C-8 hydroxyl group on C-11, leading to formation of a tetrahydrofuran ring bridging C-8 and C-11. Concurrent dehydration of the C-12 hydroxyl group generated a terminal double bond at C-12/C-20, ultimately affording compound **2**. In intermediate **b**, nucleophilic attack by the C-16 hydroxyl on C-2 resulted in cyclization to form a five-membered oxygen-containing ring. Simultaneously, the C-2 hydroperoxy group attacked C-12, establishing a peroxide bridge between C-2 and C-12. Dehydration of the C-11 hydroxyl group introduced a double bond between C-10 and C-11, yielding compound **1**. Intermediate **e** was transformed through a sequence of dehydration and oxidation steps to generate compounds **7** and **3**. Oxidation of intermediate **f** produced compound **6**. Subsequent C-7/C-8 ring-opening, when followed by dehydration or methylation, afforded compounds **4** or **5**, respectively (Scheme 1). It is noteworthy that the proposed cyclization follows Baldwin’s rules, with the 5-exo-tetr process being both stereoelectronically allowed and kinetically favored.

### 2.3. PPARs Agonistic Activity of Compounds **1**–**7**

**Figure 6:**
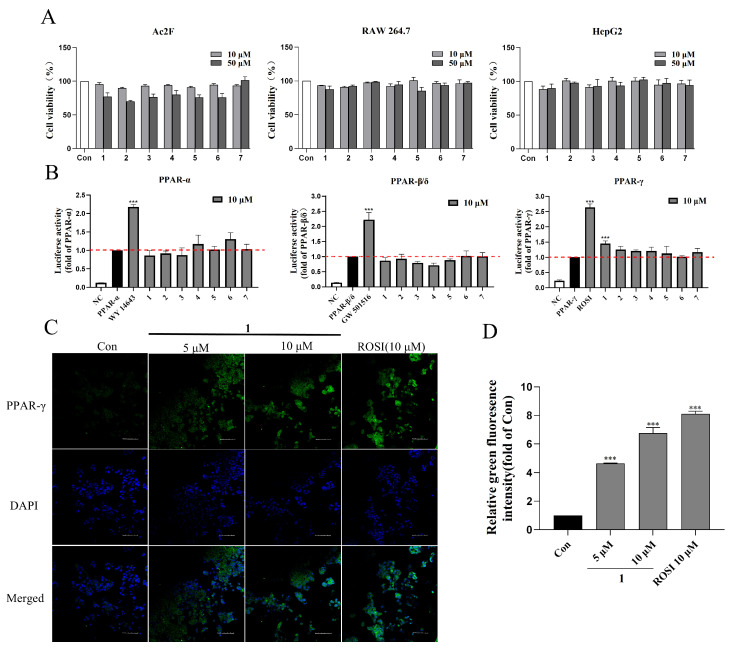
The effects of compounds **1**–**7** on the cell viability and transcriptional activation of PPARs. (**A**) Effects of compounds on Ac2F, RAW 264.7, and HepG2 cell viability; (**B**) The PPAR-α/β/γ transcriptional activity of the compounds were evaluated by luciferase assay. WY14643, GW501516, and rosiglitazone (ROSI) were used as positive control for PPAR-α, PPAR-β/δ, and PPAR-γ activation, respectively. (**C**) PPAR-γ was visualized using confocal microscopy as green fluorescence and the cell nucleus was viewed as cyan fluorescence by DAPI staining. (**D**) Quantification of PPAR-γ expression. *** p < 0.001 vs. PPAR-α, -β/δ or -γ group. The scale bar represents 100 μm.

In bioassays, the cell viability of rat liver Ac2F cells, mouse macrophages RAW264.7, and human hepatoma HepG2 cells was evaluated by MTT assay after compounds treatment for 24 h. Compounds **1**–**7** (10 μM and 50 μM) showed no significant cytotoxicity, maintaining over 90% cell viability in all three cell lines (Figure 6A). The transcriptional effects of compounds **1**–**7** on PPARs were assessed using a luciferase assay. The agonists WY14643, GW501506, and rosiglitazone (ROSI) were employed as positive controls for PPAR-α, PPAR-β/δ, and PPAR-γ activation, respectively. As shown in Figure 6B, compound **1** at 10 μM exhibited significant and selective agonist activity toward PPAR-γ, with no notable activation of the PPAR-α or PPAR-β/δ subtypes (Figure 6B). Immunofluorescence analysis revealed that compound **1** (5 and 10 μM, 6 h) concentration-dependently enhanced PPAR-γ expression in HepG2 cells, as indicated by green fluorescence labeling (Figure 6C). Treatment with 10 μM rosiglitazone resulted in an 8.11-fold upregulation of PPAR-γ expression relative to the control. In comparison, compound **1** at 5 μM and 10 μM promoted PPAR-γ expression by 4.65- and 6.76-fold, respectively. This dose-dependent response indicates that the activity of compound **1** is comparable to that of the positive control rosiglitazone.

**Figure 7:**
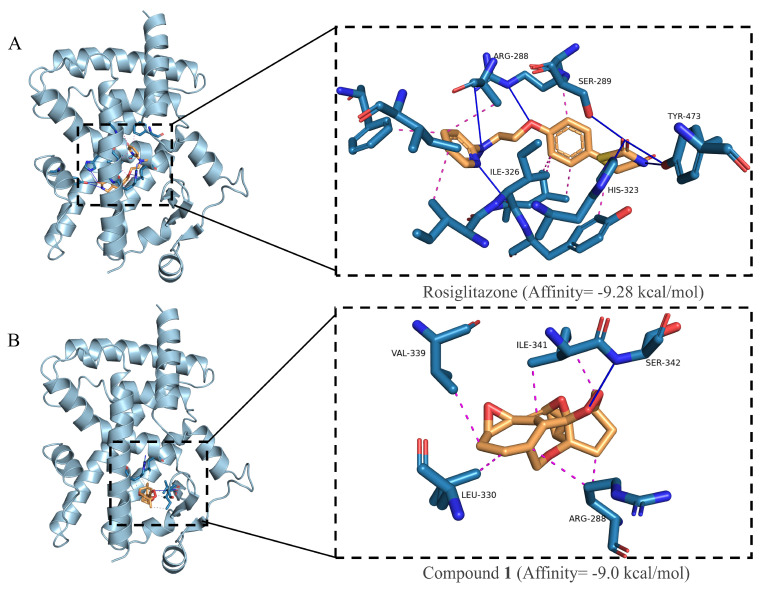
Docking simulation of ligand and PPAR-γ (PDB ID:4EMA) interaction. (**A**) Docking pose and binding interactions of rosiglitazone with PPAR-γ. (**B**) Docking pose and binding interactions of compound **1** with PPAR-γ. H-bonds were shown in blue line, hydrophobic interactions were shown in pink dotted line.

Furthermore, molecular docking simulations provided insight into the interaction of the ligands with the PPAR-γ target. Rosiglitazone occupied the ligand-binding domain (LBD) of PPAR-γ, forming hydrogen bonds with Arg288, Ser289, His323, Ile326, and Tyr473 (Figure 7A). The binding affinity score was −9.28 kcal/mol. Compound **1** occupied LBD in a shallower binding mode compared to rosiglitazone. It established a hydrogen bond with Ser342, located in the outer region of the binding cavity, and engaged in hydrophobic interactions with Arg288, Leu330, Val339, and Ile341 (Figure 7B). The binding energy for this compound was calculated to be −9.0 kcal/mol. This distinct binding mode may explain its lower potency relative to rosiglitazone at an equivalent concentration.

### 2.4. Glucose Uptake Ability of Compound **1**

**Figure 8:**
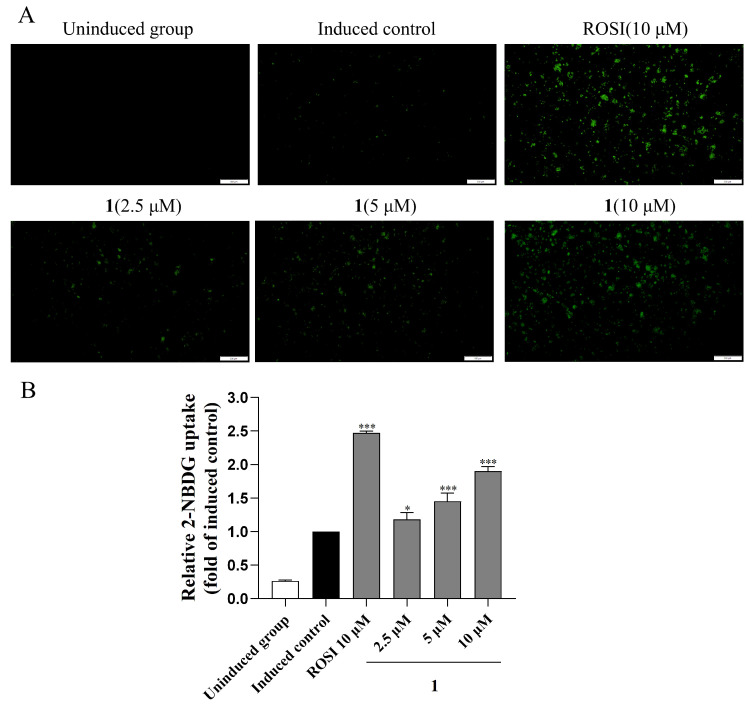
Evaluation of glucose uptake by compound **1** in HepG2 cells. (**A**) The representative fluorescence microscopic images of glucose uptake. (**B**) Quantitative analysis of glucose uptake. Fluorescence intensity was measured using a fluorescence microplate reader (Ex/Em = 460/540 nm). Rosiglitazone was employed as positive control. * p < 0.05, *** p < 0.001 vs. induced control. The scale bar represents 500 μm.

The liver plays an important role in systemic glucose homeostasis by regulating gluconeogenesis, glycogenesis, and glycolysis. PPAR-γ activity is crucial for maintaining this glucose balance. As detailed in Figure 8, following a 24 h period of serum-free culture and glucose deprivation, the cells were treated with either rosiglitazone or compound **1**. Subsequent incubation with or without the fluorescent glucose analog 2-NBDG for 90 min revealed that compound **1** promoted glucose uptake in a dose-dependent manner, showing 1.18-, 1.45-, and 1.90-fold increases at concentrations of 2.5, 5, and 10 μM, respectively, compared with the induced control. As expected for the positive control, rosiglitazone also significantly enhanced 2-NBDG uptake, producing a 2.47-fold increase relative to the control group.

### 2.5. Adipogenesis and Lipid Accumulation Effects of Compounds **1**

**Figure 9:**
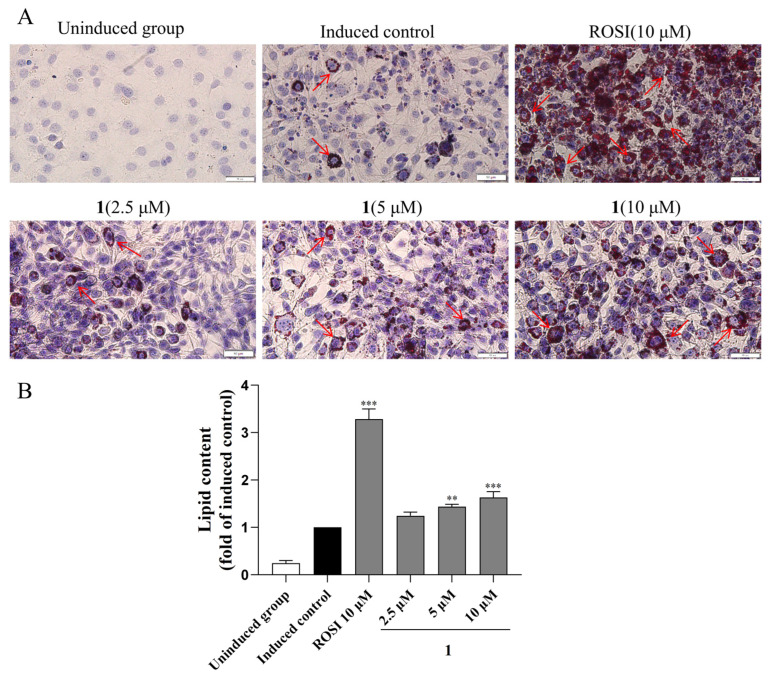
Effect of compound **1** on adipocyte differentiation and lipid accumulation in 3T3-L1 cells. (**A**) Morphology of 3T3-L1 after adipocyte differentiation with treatment of rosiglitazone and compound **1**. Cells were stained with Oil Red O. Red droplets represent the lipid contents. (**B**) Quantitative analysis of lipid accumulation. The stained cells were washed with isopropanol, and the absorbance was measured at 510 nm using a microplate reader. The scale bar represents 50 μm. ** p < 0.01, *** p < 0.001 vs. induced control.

In adipose tissue, PPAR-γ activation promotes adipocyte differentiation, leading to enhanced glucose uptake via the formation of insulin-sensitive adipocytes. However, this beneficial effect is often accompanied by adverse effects such as fat accumulation and weight gain. To assess the adipogenic activity of compound **1**, we evaluated its effect on 3T3-L1 adipocyte differentiation by measuring lipid accumulation via Oil Red O staining. Following the induction of differentiation in the presence of the rosiglitazone (10 μM), mature adipocytes with intracellular lipid droplets-stained red were observed, as indicated by the red arrows in Figure 9A. Notably, compound **1** at 10 μM induced mild lipid accumulation (1.63-fold versus induced control), which was substantially lower than the 3.28-fold increase observed with rosiglitazone at the same concentration (Figure 9). Collectively, our findings demonstrate that compound **1**, a selective PPAR-γ agonist, enhances glucose uptake in hepatic cells while reducing adipogenesis and lipid accumulation. The favorable activity and safety profile of compound **1** highlights its potential as a lead structure for developing novel anti-diabetic agents.

## 3. Materials and Methods

### 3.1. General Experimental Procedures

Melting points were determined using an X-4 digital micro-melting point apparatus (Shanghai INESA Physico-Optical Instrument Co., Ltd., Shanghai, China). Single-crystal X-ray diffraction data were collected on a Bruker D8 Venture diffractometer (Bruker Biospin AG, Fällanden, Switzerland) equipped with Cu K*α* radiation. Optical rotations were measured on a PerkinElmer 241 MC polarimeter (Perkin Elmer, Springfield, IL, USA). IR spectra were recorded on a Nicolet iS50 spectrometer (Thermo Fisher Scientific, Madison, WI, USA). ^1^H and ^13^C NMR spectra were obtained on a Bruker DRX-600 spectrometer (Bruker Biospin AG, Fällanden, Switzerland), with chemical shifts referenced to residual CDCl_3_ (*δ*_H_ 7.26 ppm) for ^1^H NMR and CDCl_3_ (*δ*_C_ 77.16 ppm) for ^13^C NMR. HR-ESIMS data were acquired on a ZenoTOF 7600 mass spectrometer (AB Sciex Pte. Ltd., Singapore). Column chromatography was carried out on silica gel (200–300 and 300–400 mesh, Qingdao Haiyang Chemical Co., Ltd., Qingdao, China) and Sephadex LH-20 (Amersham™, Little Chalfont, UK). Analytical TLC was performed on precoated silica gel plates (G60 F-254, Yan Tai Zhi Fu Chemical Group Co., Yantai, China), with visualization under UV light or by heating after spraying with anisaldehyde–H_2_SO_4_ reagent. RP-HPLC was conducted on an Agilent 1260 series LC system (Agilent Technologies, Santa Clara, CA, USA) equipped with a DAD G1315D detector at 210 nm (Agilent, Santa Clara, CA, USA), employing a semi-preparative Agilent XDB-C18 column (5 μm, 250 × 9.4 mm) for purification. Solvents used for column chromatography and HPLC were of analytical grade (Shanghai Chemical Reagents Co., Ltd., Shanghai, China) and chromatographic grade (Dikma Technologies Inc., Beijing, China), respectively. The following antibodies were used: PPAR-γ (Santa Cruz Biotechnology, Santa Cruz, CA, USA), and GAPDH (proteintech, Shanghai, China). Anti-rabbit horseradish-linked IgG was used as the secondary antibody (Immunoway, Plano, TX, USA). Dulbecco’s Modified Eagle Medium (DMEM) and fetal bovine serum (FBS) were purchased from Gibco (from Thermo Fisher Scientific, Waltham, MA, USA). Penicillin–streptomycin–gentamicin solution (Solarbio Science & Technology Co., Ltd., Beijing, China), MTT reagent, rosiglitazone (Adamas-beta, Shanghai, China), WY14643, GW501516, IBMX and 2-NBDG (MCE, Monmouth Junction, NJ, USA) were commercially available reagents.

### 3.2. Animal Materials

The soft coral was collected in 2019 at a depth of 20 m near Ximao Island, Hainan Province, China, and identified as *S. crassocaule* by Prof. Xiubao Li of Hainan University. The specimen (No. 19-XD-32) has been deposited at Shandong Laboratory of Yantai Drug Discovery, Bohai Rim Advanced Research Institute for Drug Discovery.

### 3.3. Extraction and Isolation

The frozen animals (350 g, dry weight after extraction) were cut into small pieces and exhaustively extracted with acetone at room temperature (4 × 2 L). The combined acetone extracts were evaporated to dryness, yielding a brown residue, which was subsequently partitioned between diethyl ether (Et_2_O) and water. The Et_2_O layer was concentrated under reduced pressure to afford a dark brown residue (18 g). This material was subjected to silica gel column chromatography (200–300 mesh) and eluted with a gradient of petroleum ether (PE)/Et_2_O (0–100%), giving ten fractions (A–J).

Fraction H (1.1 g) was subjected to Sephadex LH-20 column chromatography using a PE/CH_2_Cl_2_/MeOH (2:1:1) eluent, affording subfractions HA–HD. The HD subfraction was further purified over Sephadex LH-20 (CH_2_Cl_2_) and silica gel column chromatography (PE/Et_2_O, 30:1), resulting in four subfractions HD1–HD5. Subsequent purification of HD2 by RP-HPLC [CH_3_CN/H_2_O (70:30), 3.0 mL/min] yielded compounds **6** (5 mg, t_R_ = 18 min) and **7** (3 mg, t_R_ = 23 min), whereas HD5 was purified by RP-HPLC [CH_3_CN/H_2_O (68:32), 3.0 mL/min] to afford compound **5** (2 mg, t_R_ = 14 min). Fraction I (500 mg) was purified on Sephadex LH-20 as described above, producing subfractions IDA–IDD. Further purification of IDC by semi-preparative RP-HPLC (CH_3_CN/H_2_O, 55:45) gave compounds **2** (2.5 mg, t_R_ = 20 min) and **4** (1.5 mg, t_R_ = 22.5 min). Fraction J (1.2 g) was similarly purified by Sephadex LH-20 to yield subfractions J3A–J3E, and semi-preparative RP-HPLC of J3K (CH_3_CN/H_2_O, 47:53) afforded compounds **1** (1.2 mg, t_R_ = 13 min) and **3** (2 mg, t_R_ = 21 min).

### 3.4. Spectroscopic Data of Compounds

Sarcocraol A (**1**): Colorless crystal, (mp 164–169 °C), ${\left[\alpha \right]}_{D}^{20}$ +24 (c 0.10, MeOH); IR (KBr): ν_max_ 2919, 2856, 1460, 1371 cm^−1^; For ^1^H and ^13^C NMR spectroscopic data, see Table 1; HR-ESIMS *m*/*z* 349.2004 ([M + H]^+^, calcd for 349.2009).

Sarcocraol B (**2**): Colorless oil, ${\left[\alpha \right]}_{D}^{20}$ +13 (c 0.10, MeOH); IR (KBr): ν_max_ 3483, 2925, 2863, 1700, 1208 cm^−1^; For ^1^H and ^13^C NMR spectroscopic data, see Table 1; HR-ESIMS *m*/*z* 319.2265 ([M + H]^+^, calcd 319.2268).

### 3.5. Calculation Section

For the QM-NMR calculations, conformational searches were conducted in MacroModel 9.9.223 (Schrödinger, http://www.schrodinger.com/MacroModel, accessed on 15 March 2025) using the torsional Monte Carlo multiple minimum (MCMM) method combined with the OPLS_2005 force field, within an energy cutoff of 21 kJ/mol (5.02 kcal/mol). Conformers representing more than 1% of the Boltzmann population were further reoptimized at the B3LYP/6-31G (d, p) level. Subsequent NMR shielding constants were obtained at the PCM/mPW1PW91/6-311+G (d, p) level in Gaussian 09 [26], employing the GIAO approach [21,22,23]. The Boltzmann-weighted average shielding values for each stereoisomer were then calculated and compared with the experimental spectra. For the TDDFT-ECD calculations, the conformational search protocol was consistent with that used for the QM-NMR study. Conformers with populations above 1% were reoptimized and subjected to TDDFT-ECD calculations in Gaussian 09 at the B3LYP/6-311G (d, p) level with the IEFPCM solvent model. The resulting spectra were generated and analyzed using SpecDis 1.71 software [24].

### 3.6. X-Ray Crystallographic Analysis for Compound **1**

The crystallographic data were collected on a Bruker D8 Venture diffractometer equipped with Cu K*α* radiation (λ = 1.54178 Å). The structures were solved with the ShelXT structure solution program using Intrinsic Phasing and refined with the ShelXL refinement package using Least Squares minimisation.

Compound **1**: colorless crystals (mp 164–169 °C), orthorhombic, C_20_H_28_O_5_, Mr = 348.42, crystal size 0.2 × 0.02 × 0.02 mm^3^, space group P2_1_2_1_2_1_, a = 11.3335(5) Å, b = 11.5001(5) Å, c = 14.1664(6) Å, V = 1846.40(14) Å3, Z = 4, ρcalc = 1.253 g/cm^3^, F (000) = 752.0, Independent reflections: 3807 [R_int_ = 0.0775, R_sigma_ = 0.0371]. R_1_ = 0.0390, wR_2_ = 0.0961 reflections with I ≥ 2σ (I), R_1_ = 0.0528, wR_2_ = 0.1050 for all unique data, Flack parameter: −0.08(10). The crystals of **1** were recrystallized from MeOH. These above crystal data were deposited in the Cambridge Crystallographic Data Centre (CCDC) and assigned the accession number (CCDC 2428505).

### 3.7. Cell Culture and Cell Viability

Rat liver Ac2F cell line was obtained from the American Type Culture Collection (ATCC, Rockville, MD, USA). Mouse adipocyte 3T3-L1 cell line and human hepatoma HepG2 cell line were purchased from National Collection of Authenticated Cell Cultures of China. These cells maintained in Dulbecco’s Modified Eagle Medium (DMEM) containing 2 mM L-glutamine, 100 μg/mL streptomycin, 2.5 μg/mL amphotericin B, and 10% fetal bovine serum (FBS), under a humidified atmosphere of 5% CO_2_ at 37 °C. For cytotoxicity assay, cells were seeded into 96-well plates at a density of 1 × 10^4^ cells/well and allowed to adhere for 12 h. Cells were then exposed to test compounds **1**–**7** and cultured in serum-free medium for 24 h. Following treatment, 20 μL of MTT solution (3-(4,5-dimethylthiazol-2-yl)-2,5-diphenyltetrazolium bromide, 5 mg/mL) was added to each well and incubated at 37 °C for 4 h in the dark. The supernatant was then carefully aspirated, and formazan crystals were solubilized by adding 150 μL dimethyl sulfoxide (DMSO) per well. The absorbance was quantified at 490 nm using a microplate spectrophotometer (EnVision, PerkinElmer, Pontyclun, UK).

### 3.8. Luciferase Assay

For the luciferase assays, the 3×AOX-TK-luciferase reporter plasmid containing three copies of the PPRE from the acyl-CoA oxidase promoter was provided by Dr. Christopher K. Glass (University of California, San Diego, CA, USA). The pcDNA3 vector and full-length human PPAR-α, -β/δ, or -γ expression plasmids (pFlag-PPAR-α/β/γ) were gifts from Dr. Chatterjee (University of Cambridge, Cambridge, UK). For transfection, Ac2F cells (1 × 10^5^ cells/well in 48-well plates) were co-transfected with 1 μg of reporter PPRE plasmid and 0.1 μg of effector plasmid (pcDNA3 or pFlag-PPAR-α/β/γ) using LipofectamineTM 2000 in serum-free medium. After 6 h of transfection, the medium was replaced with complete medium containing 10% FBS, and the cells were cultured for an additional 12 h. Subsequently, the medium was replaced with serum-free medium containing the test compounds. Following 7 h of incubation, luciferase activity was measured using the Luciferase Assay System (Promega) with a microplate reader. (EnVision, PerkinElmer, Pontyclun, UK).

### 3.9. Immunofluorescence

HepG2 cells were seeded in confocal dishes and allowed to reach 80% confluence. Then, the cells were incubated with test compounds for 6 h. Subsequently, the cells were fixed with 4% paraformaldehyde for 15 min, washed three times with phosphate-buffered saline (PBS), and permeabilized with 0.5% (*v*/*v*) Triton X-100/PBS for 15 min. After another three washes with PBS, the cells were blocked with 10% FBS/PBS at room temperature for 30 min. The cells were then incubated overnight at 4 °C with a primary antibody against PPAR-γ (Upingbio, Shanghai, China), washed three times with PBS, and incubated at room temperature for 30 min with an Alexa Fluor 488-conjugated anti-rabbit secondary antibody (Immunoway, Plano, TX, USA). Following three additional PBS washes, the cells were stained with DAPI (5 mg/mL) at room temperature for 20 min. Finally, the samples were observed under a confocal microscope (Olympus FV3000, Tokyo, Japan) using an excitation wavelength of 488 nm and an emission wavelength of 519 nm.

### 3.10. Molecular Docking

The crystal structure of PPAR-γ (PDB ID: 4EMA) was obtained from the RCSB Protein Data Bank and preprocessed using PyMOL version 3.9 software for the addition of hydrogen atoms and the removal of water molecules. Subsequently, the molecular structures of rosiglitazone and compound **1** were converted into PDB format, and molecular docking analysis was performed using AutoDock version 1.5.7 software. The final docking results were visualized using PyMOL3.9.

### 3.11. Glucose Uptake Assay

HepG2 cells were plated in 96-well plates and cultured overnight in complete medium (DMEM supplemented with 10% FBS). After 24 h incubation in glucose-free medium (DMEM/F-12 containing 10% FBS), the cells were treated with either rosiglitazone (10 μM) or compound **1** (2.5, 5, and 10 μM), in the presence or absence of 2-NBDG (50 μM). Following 90 min of drug treatment, the cells were washed with phosphate-buffered saline (PBS) and visualized under a fluorescence microscope using an excitation wavelength of 460 nm and an emission wavelength of 540 nm.

### 3.12. Adipocyte Differentiation Assay

3T3-L1 preadipocytes were seeded in 48-well plates at a density of 1 × 10^5^ cells per well and cultured until reaching confluence. Compound **1** (2.5, 5, and 10 μM) or rosiglitazone (10 μM) were added to MDI differentiation medium (DMEM + 10% FBS + 0.5 mM IBMX + 0.25 μM dexamethasone + 1 μg/mL insulin), and the cells were cultured in this medium for 48 h. Subsequently, the cells were cultured in insulin-containing maintenance medium (DMEM + 10% FBS + 1 μg/mL insulin) supplemented with the respective compounds for an additional 48 h. Thereafter, the cells were treated with same samples using complete medium (DMEM + 10% FBS) for another 4 days, with the medium refreshed every 2 days. After the differentiation protocol, the supernatant was removed, and cells were washed with PBS and fixed with paraformaldehyde for 30 min. Fixed cells were stained with Oil Red O for 1 h at room temperature and examined under a microscope for qualitative analysis. For quantitative analysis, the stained lipid droplets were eluted using isopropanol, and the absorbance at 510 nm was measured using a microplate multimode reader (Olympus, IX73, Tokyo, Japan).

### 3.13. Statistical Analysis

All biological experiments were performed in triplicate and presented as mean ± standard error of the mean (SEM). Intergroup differences were determined by one-way analysis of variance (ANOVA). Statistical significance was defined as * p < 0.05, ** p < 0.01, and *** p < 0.001.

## 4. Conclusions

In conclusion, two new highly oxygenated cembrane-type diterpenes, along with five known analogues, were isolated from the soft coral *S. crassocaule*. Their structures were unambiguously determined through comprehensive spectroscopic analysis, computational methods, and X-ray crystallography, thereby enriching the structural diversity of cembrane diterpenes. Possible biosynthetic pathway of all isolated compounds was hypothesized. The new compound (**1**) demonstrated selective PPAR-γ agonist activity, promoting concentration-dependent glucose uptake in HepG2 cells without inducing excessive adipogenic differentiation in 3T3-L1 preadipocytes. These properties highlight its potential as a promising lead for the development of antidiabetic agents.

## Figures and Tables

**Table 1:** ^1^H (600 MHz) and ^13^C NMR (150 MHz) data of compounds **1** and **2** in CDCl_3_.

No.	1		2	
	*δ*_H_ Mult. (*J* in Hz)	*δ*_C_, Type	*δ*_H_ Mult. (*J* in Hz)	*δ*_C_, Type
1		65.72, qC		133.24, qC
2		112.39, qC	5.45 brs	85.35, CH
3	5.37 brs	117.80, CH	5.20 d (9.62)	126.80, CH
4		143.57, qC		139.56, qC
5	2.23 m	36.38, CH_2_	2.28 m	37.19, CH_2_
	2.34 m		2.28 m	
6	2.05 m	26.08, CH_2_	1.33 m	28.08, CH_2_
	1.73 m		2.15 m	
7	2.64 dd (7.31, 3.32)	61.58, CH	4.34 d (9.15)	67.85, CH
8		61.83, qC		80.74, qC
9	2.77 dd (12.23, 3.46)	44.27, CH_2_	1.59 m	30.03, CH_2_
	1.76 m		2.23 m	
10	5.50 m	123.43, CH	1.50 m	25.90, CH_2_
			2.10 m	
11	5.64 d (16.08)	135.97, CH	4.63 dd (12.17, 3.13)	84.52, CH
12		86.53, qC		147.7, qC
13	1.76 m	32.67, CH_2_	1.95 m	30.76, CH_2_
	2.16 m		2.23 m	
14	1.74 m	23.52, CH_2_	2.20 m	27.49, CH_2_
	2.60 m		2.06 m	
15		68.75, qC		128.50, qC
16	3.97 d (9.81)	71.05, CH_2_	4.48 d (11.91)	78.51, CH_2_
	4.07 d (9.81)		4.56 dd (11.82 5.20)	
17	1.68 s	12.39, CH_3_	1.68 s	10.39, CH_3_
18	1.93 s	19.44, CH_3_	1.83 s	15.91, CH_3_
19	1.41 s	17.74, CH_3_	1.03 s	18.78, CH_3_
20	1.22 s	26.99, CH_3_	4.96 s	115.00, CH_2_
			5.00 s	

## Data Availability

The data that support the findings of this study are available in the Supplementary Material of this article.

## Supplementary Materials

The following supporting information can be downloaded at: https://www.mdpi.com/article/10.3390/md23120450/s1, Section S1: Spectra of compound **1**; Section S2: Spectra of compound **2**; Section S3: Ouantum chemical calculations of NMR shifts for compound **2**.

## References

[1] Berger J., Moller D.E. (2002). The Mechanisms of Action of PPARs. Annu. Rev. Med..

[2] Grygiel-Górniak B. (2014). Peroxisome proliferator-activated receptors and their ligands: Nutritional and clinical implications—A review. Nutr. J..

[3] Montaigne D., Butruille L., Staels B. (2021). PPAR control of metabolism and cardiovascular functions. Nat. Rev. Cardiol..

[4] Francque S., Szabo G., Abdelmalek M.F., Byrne C.D., Cusi K., Dufour J.-F., Roden M., Sacks F., Tacke F. (2021). Nonalcoholic steatohepatitis: The role of peroxisome proliferator-activated receptors. Nat. Rev. Gastroenterol. Hepatol..

[5] Singh A., Chaudhary R. (2025). Potentials of peroxisome proliferator-activated receptor (PPAR) α, β/δ, and γ: An in-depth and comprehensive review of their molecular mechanisms, cellular Signalling, immune responses and therapeutic implications in multiple diseases. Int. Immunopharmacol..

[6] Huang J.V., Greyson C.R., Schwartz G.G. (2012). PPAR-γ as a therapeutic target in cardiovascular disease: Evidence and uncertainty. J. Lipid Res..

[7] Yamazaki T., Cable E.E., Schnabl B. (2025). Peroxisome proliferator–activated receptor delta and liver diseases. Hepatol. Commun..

[8] Naim M.J., Alam O., Alam M.J., Shaquiquzzaman M., Alam M.M., Naidu V.G.M. (2018). Synthesis, Docking, In Vitro and In Vivo Antidiabetic Activity of Pyrazole Based 2,4-Thiazolidinedione Derivatives as PPAR-γ Modulators. Arch. Pharm..

[9] Zhang C., Yu H., Ye J., Tong H., Wang M., Sun G. (2023). Ginsenoside Rg3 Protects against Diabetic Cardiomyopathy and Promotes Adiponectin Signaling via Activation of PPAR-γ. Int. J. Mol. Sci..

[10] Jiang H., Zhou X.E., Shi J., Zhou Z., Zhao G., Zhang X., Sun Y., Suino-Powell K., Ma L., Gao H. (2020). Identification and structural insight of an effective PPARγ modulator with improved therapeutic index for anti-diabetic drug discovery. Chem. Sci..

[11] D’Aniello E., Amodeo P., Vitale R. (2023). Marine Natural and Nature-Inspired Compounds Targeting Peroxisome Proliferator Activated Receptors (PPARs). Mar. Drugs.

[12] Quang T.H., Ha T.T., Minh C.V., Kiem P.V., Huong H.T., Ngan N.T.T., Nhiem N.X., Tung N.H., Thao N.P., Thuy D.T.T. (2011). Cytotoxic and PPARs transcriptional activities of sterols from the Vietnamese soft coral *Lobophytum laevigatum*. Bioorg. Med. Chem. Lett..

[13] Weng J.-R., Chiu C.-F., Hu J.-L., Feng C.-H., Huang C.-Y., Bai L.-Y., Sheu J.-H. (2018). A Sterol from Soft Coral Induces Apoptosis and Autophagy in MCF-7 Breast Cancer Cells. Mar. Drugs.

[14] Ke L.-M., Yu D.-D., Su M.-Z., Cui L., Guo Y.-W. (2024). In Vitro Insights into the Role of 7,8-Epoxy-11-Sinulariolide Acetate Isolated from Soft Coral *Sinularia siaesensis* in the Potential Attenuation of Inflammation and Osteoclastogenesis. Mar. Drugs.

[15] Hsu C.-M., Lin J.-J., Su J.-H., Liu C.-I. (2022). 13-Acetoxysarcocrassolide induces apoptosis in human hepatocellular carcinoma cells through mitochondrial dysfunction and suppression of the PI3K/AKT/mTOR/p70S6K signalling pathway. Pharm. Biol..

[16] Saleh H.A., Raafat K.M., Temraz T.A., Noureldin N., Breitinger H.-G., Breitinger U. (2020). Sarcophine and (7S, 8R)-dihydroxydeepoxysarcophine from the Red Sea soft coral *Sarcophyton glaucum* as In Vitro *and* In Vivo Modulators of Glycine Receptors. Neurotoxicology.

[17] Sun M., Li S., Zeng J., Guo Y., Wang C., Su M. (2024). Two New Diterpenoids Formed by Transannular Diels–Alder Cycloaddition from the Soft Coral *Sarcophyton tortuosum,* and Their Antibacterial and PPAR-β Agonist Activities. Mar. Drugs.

[18] El Sayed K.A., Hamann M.T., Waddling C.A., Jensen C., Lee S.K., Dunstan C.A., Pezzuto J.M. (1998). Structurally Novel Bioconversion Products of the Marine Natural Product Sarcophine Effectively Inhibit JB6 Cell Transformation. J. Org. Chem..

[19] Hegazy M.-E.F., Mohamed T.A., Abdel-Latif F.F., Alsaid M.S., Shahat A.A., Paré P.W. (2013). Trochelioid A and B, new cembranoid diterpenes from the Red Sea soft coral *Sarcophyton trocheliophorum*. Phytochem. Lett..

[20] Hegazy M.E., El-Beih A.A., Moustafa A.Y., Hamdy A.A., Alhammady M.A., Selim R.M., Abdel-Rehim M., Paré P.W. (2011). Cytotoxic cembranoids from the Red Sea soft coral *Sarcophyton glaucum*. Nat. Prod. Commun..

[21] Wolinski K., Hinton J.F., Pulay P. (1990). Efficient implementation of the gauge-independent atomic orbital method for NMR chemical shift calculations. J. Am. Chem. Soc..

[22] Li S.-W., Cuadrado C., Yao L.-G., Daranas A.H., Guo Y.-W. (2020). Quantum Mechanical–NMR-Aided Configuration and Conformation of Two Unreported Macrocycles Isolated from the Soft Coral *Lobophytum* sp.: Energy Calculations versus Coupling Constants. Org. Lett..

[23] Ditchfield R. (1972). Molecular Orbital Theory of Magnetic Shielding and Magnetic Susceptibility. J. Chem. Phys..

[24] Mándi A., Kurtán T. (2019). Applications of OR/ECD/VCD to the structure elucidation of natural products. Nat. Prod. Rep..

[25] Shen S.-M., Li S.-W., Su M.-Z., Yao L.-G., Appendino G., Guo Y.-W. (2023). Structurally Diverse Diterpenoids from the Sanya Bay *Nudibranch Hexabranchus sanguineus* and Its Sponge-Prey *Chelonaplysilla* sp. *Chem*. Eur. J..

[26] Frisch M.J., Trucks G., Schlegel H.B., Scuseria G.E., Robb M.A., Cheeseman J., Scalmani G., Barone V., Mennucci B., Petersson G.A. (2009). Gaussian 09.

