# Supplementary material for: Novel PPAR-γ Agonist from the Soft Coral Sarcophyton crassocaule: Modulating Glucose Uptake and Lipid Droplet Formation
Source: Mar Drugs. 2025 Nov 24;23(12):450. doi: 10.3390/md23120450 (PMC12734517; doi:10.3390/md23120450)
Supplement: Supplementary file 1 [file marinedrugs-23-00450-s001.zip › marinedrugs-3986808-supplementary.pdf]

Supporting Information

Novel PPAR-γ agonist from the Soft Coral *Sarcophyton crassocaule*: modulating glucose uptake and lipid droplet formation

Jian-Ang Zeng[1,2,†], Min Sun[2,†], Yi Qi[1,4†], Song-Wei Li[3], Li-Ting Zhang[2], Si-Min Pan[2], Yue-Wei Guo[2,3,*], Ming-Zhi Su[2,*], Hui Luo[1,*]

[1] Guangdong Engineering Technology Research Center for the Development and Utilization of Mangrove Wetland Medicinal Resources, The Key Lab of Zhanjiang for R&D Marine Microbial Resources in the Beibu Gulf Rim, and School of Ocean and Tropical Medicine, Guangdong Medical University, Zhanjiang, 524023 China;

[2] Shandong Laboratory of Yantai Drug Discovery, Bohai Rim Advanced Research Institute for Drug Discovery, Yantai 264117, China;

[3] School of Medicine, Shanghai University, Shanghai 200444, China.

[4] National Engineering Research Center for Modernization of Traditional Chinese Medicine, Guangzhou 510632, China

***** Correspondence: ywguo@simm.ac.cn (Y.-W.G.); mzsu@baridd.ac.cn (M.-Z.S.); luohui@gdmu.edu.cn (H.L.)

† These authors contributed equally to this work.

1. Spectra of compound **1** 3
Figure S1. ^{1}H NMR spectrum (600 MHz) of compound **1** in $CDCl_3$. 4
Figure S2. ^{13}C NMR spectrum (150 MHz) of compound **1** in $CDCl_3$. 4
Figure S3. HSQC spectrum (600 MHz) of compound **1** in $CDCl_3$. 5
Figure S4. HMBC spectrum (600 MHz) of compound **1** in $CDCl_3$. 5
Figure S5. ^{1}H–^{1}H COSY spectrum (600 MHz) of compound **1** in $CDCl_3$. 6
Figure S6. NOESY spectrum (600 MHz) of compound **1** in $CDCl_3$. 6
Figure S7. HR-ESI-MS spectrum of compound **1**. 7
Figure S8. IR spectrum of compound **1**. 7
Figure S9. UV and CD spectrum of compound **1**. 8
2. Spectra of compound **2** 9
Figure S10. ^{1}H NMR spectrum (600 MHz) of compound **2** in $CDCl_3$. 9
Figure S11. ^{13}C NMR spectrum (600 MHz) of compound **2** in $CDCl_3$. 9
Figure S12. DEPT spectrum (150 MHz) of compound **2** in $CDCl_3$. 10
Figure S13. HSQC spectrum (600 MHz) of compound **2** in $CDCl_3$. 10
Figure S14. HMBC spectrum (600 MHz) of compound **2** in $CDCl_3$. 11
Figure S15. ^{1}H–^{1}H COSY spectrum (600 MHz) of compound **2** in $CDCl_3$. 11
Figure S16. NOESY spectrum (600 MHz) of compound **2** in $CDCl_3$. 12
Figure S17. HR-ESI-MS spectrum of compound **2**. 12
Figure S18. IR spectrum of compound **2**. 13
Figure S19. UV and CD spectrum of compound **2**. 13
3. Ouantum chemical calculations of NMR shifts for compound **2**. 14
3.1 Structures of isomers studied for compound **2**. 14
Figure S20. Structures of isomers of compound **2**. 14
3.2. Isotropic magnetic shielding constants of compound **2**. 14

1. Spectra of compound **1**

Table S1. Crystal data for compound 1.

Identification code	cu_20250109A_0m
Empirical formula	$C_{20}H_{28}O_5$
Formula weight	348.42
Temperature/K	295.00
Crystal system	orthorhombic
Space group	$P2_12_12_1$
a/Å	11.3335(5)
b/Å	11.5001(5)
c/Å	14.1664(6)
α/°	90
β/°	90
γ/°	90
Volume/Å^3	1846.40(14)
Z	4
ρ_{calc}g/cm^3	1.253
μ/mm^{-1}	0.723
F(000)	752.0
Crystal size/mm^3	0.2 × 0.02 × 0.02
Radiation	CuKα (λ = 1.54178)
2Θ range for data collection/°	9.906 to 151.268
Index ranges	$-14 \leqslant h \leqslant 14, -14 \leqslant k \leqslant 14, -16 \leqslant l \leqslant 17$
Reflections collected	33906
Independent reflections	3807 [R_{int} = 0.0775, R_{sigma} = 0.0371]
Data/restraints/parameters	3807/0/230
Goodness-of-fit on F^2	1.053
Final R indexes [I>=2σ (I)]	R_1 = 0.0390, wR_2 = 0.0961
Final R indexes [all data]	R_1 = 0.0528, wR_2 = 0.1050
Largest diff. peak/hole / e Å^{-3}	0.13/-0.14
Flack parameter	-0.08(10)

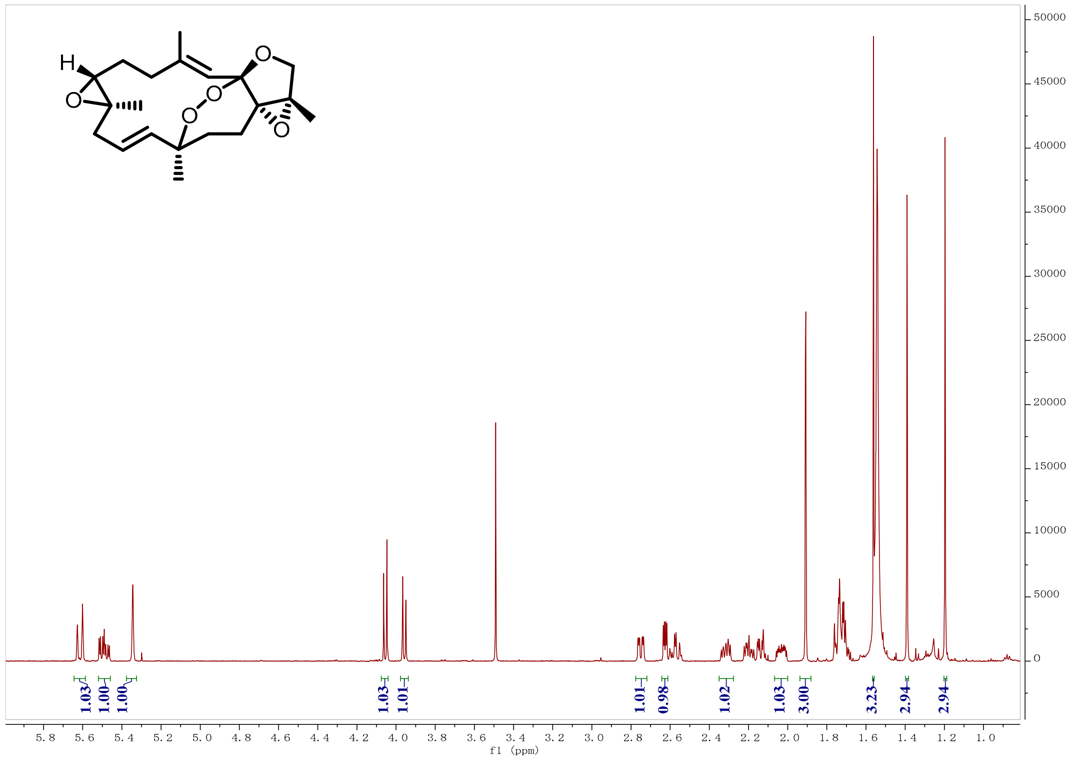

Figure S1. ^{1}H NMR spectrum (600 MHz) of compound **1** in $CDCl_3$.

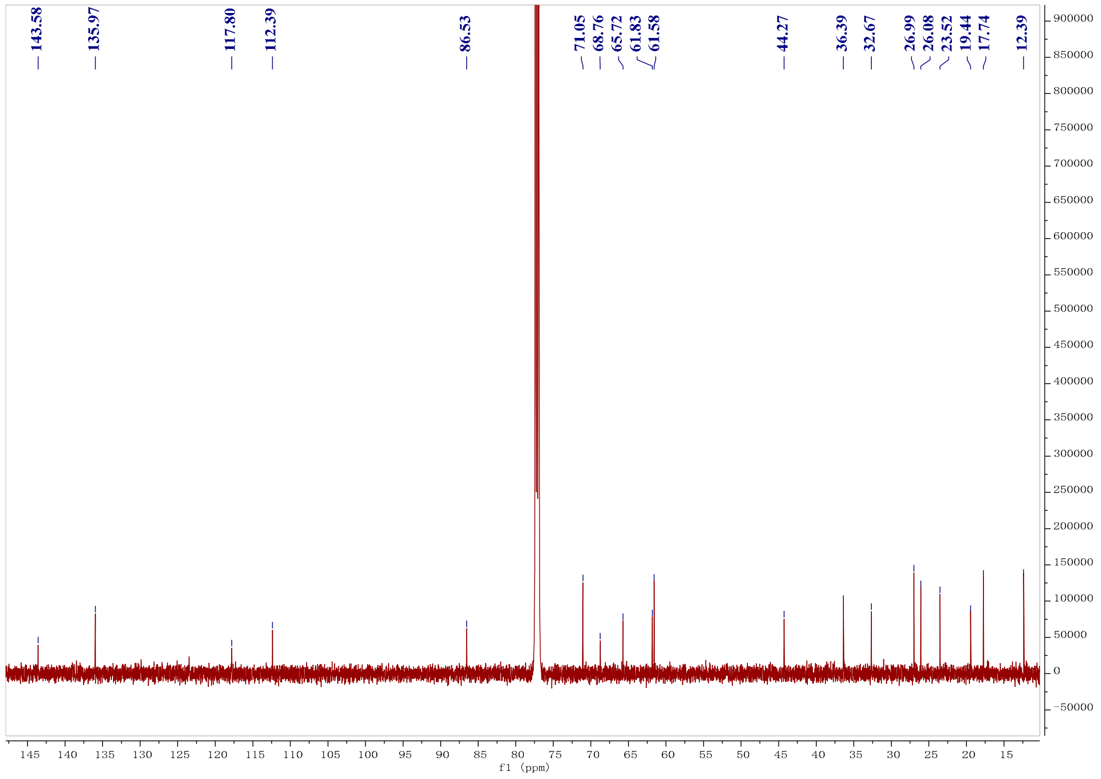

Figure S2. ^{13}C NMR spectrum (150 MHz) of compound **1** in $CDCl_3$.

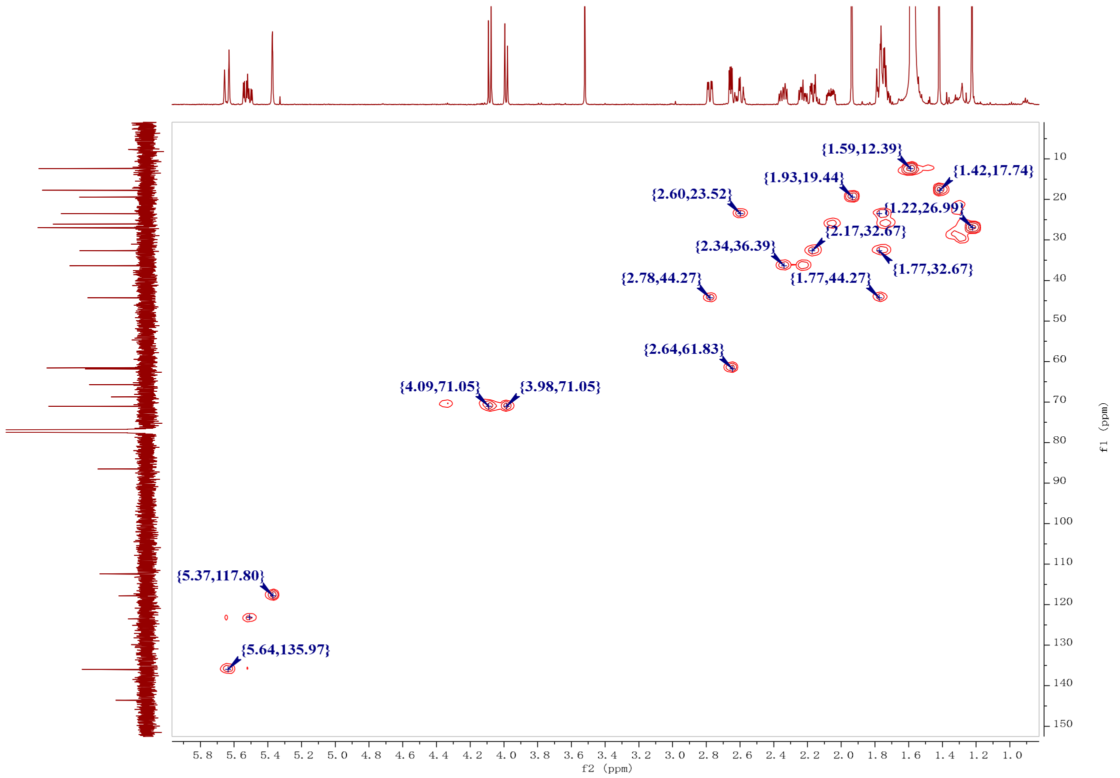

Figure S3. HSQC spectrum (600 MHz) of compound **1** in $CDCl_3$.

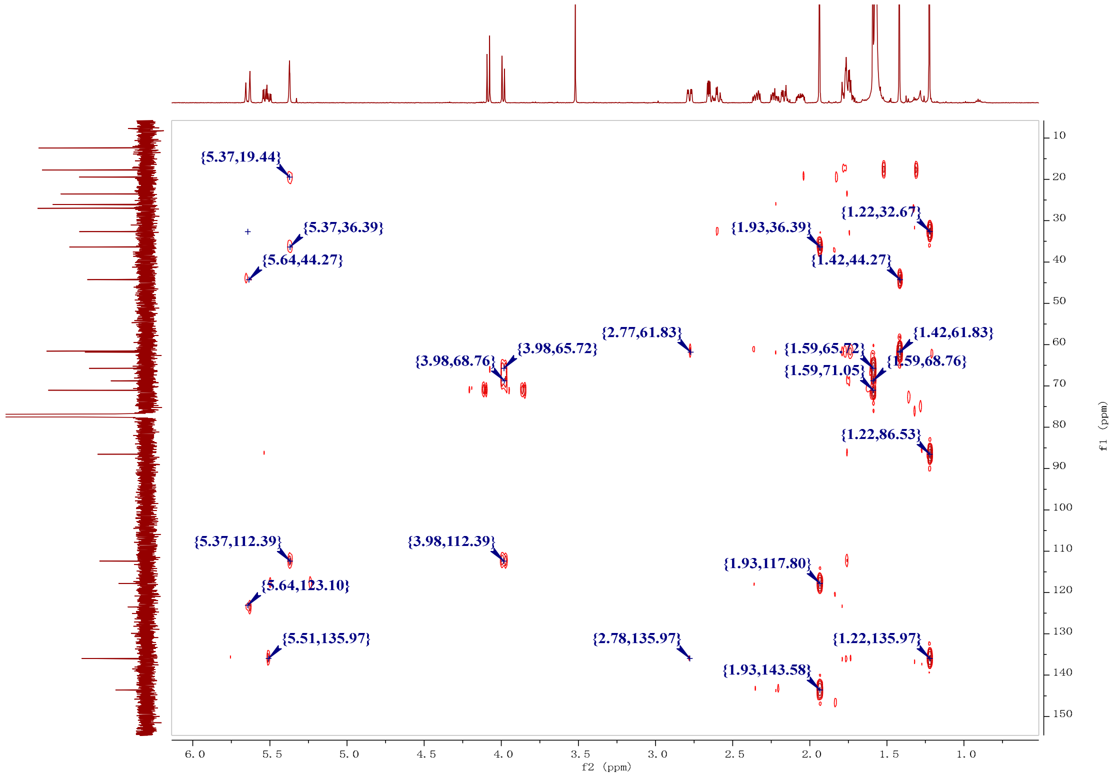

Figure S4. HMBC spectrum (600 MHz) of compound **1** in $CDCl_3$.

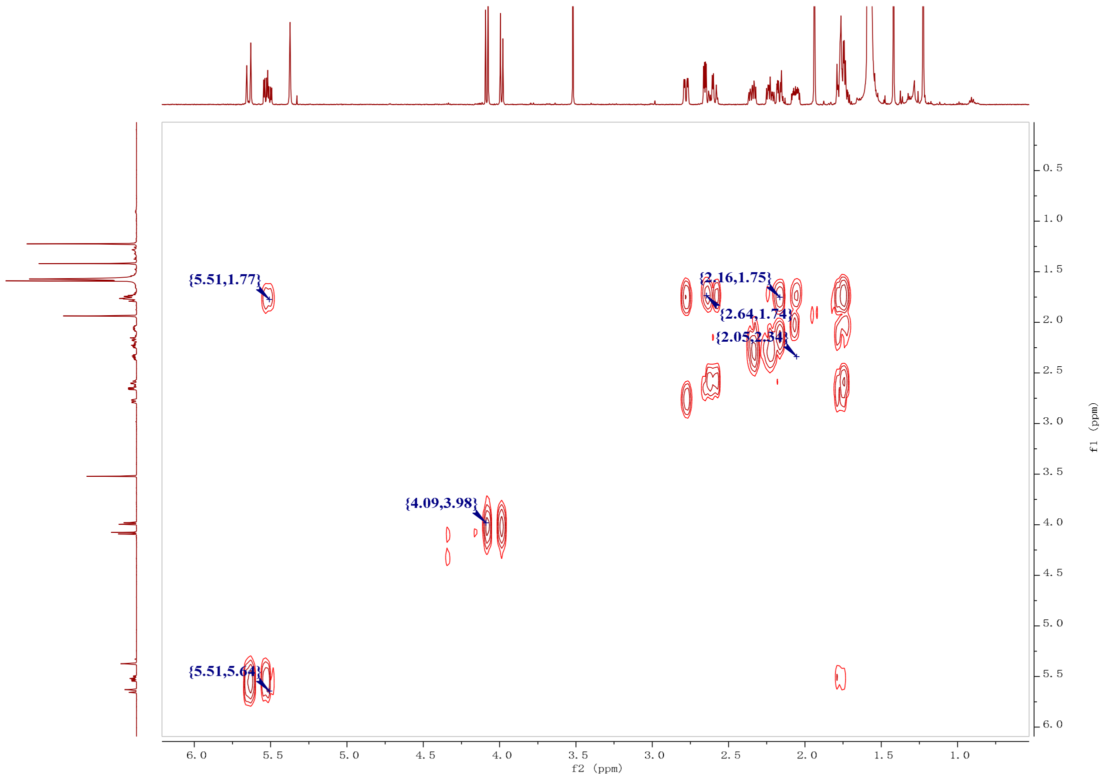

Figure S5. ^{1}H–^{1}H COSY spectrum (600 MHz) of compound **1** in $CDCl_3$.

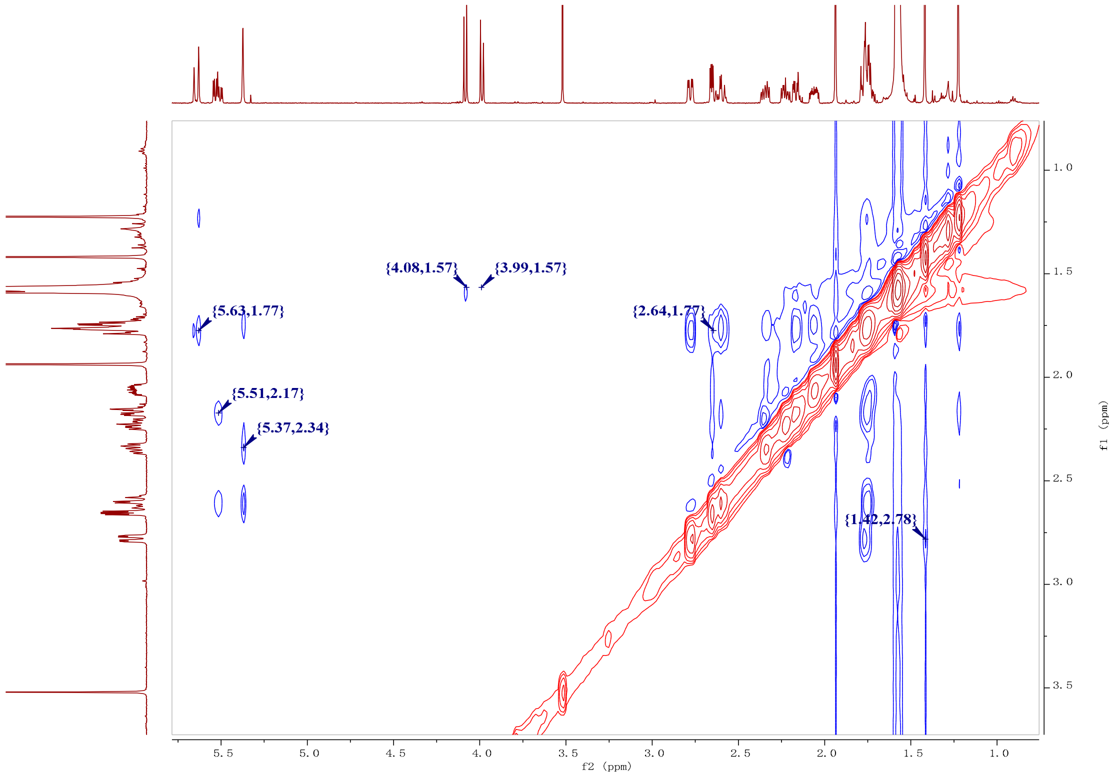

Figure S6. NOESY spectrum (600 MHz) of compound **1** in $CDCl_3$.

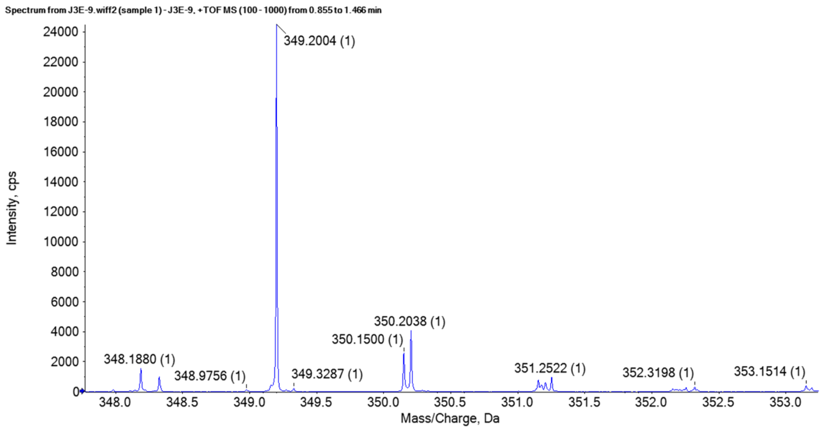

Measured m/z	Cal m/z	Error(mmu)	Error(ppm)	Ion Formula	Ion
349.2004	349.2009	-0.5	-1.6	$C_{20}H_{29}O_5$	$[M+H]^+$

Figure S7. HR-ESI-MS spectrum of compound **1**.

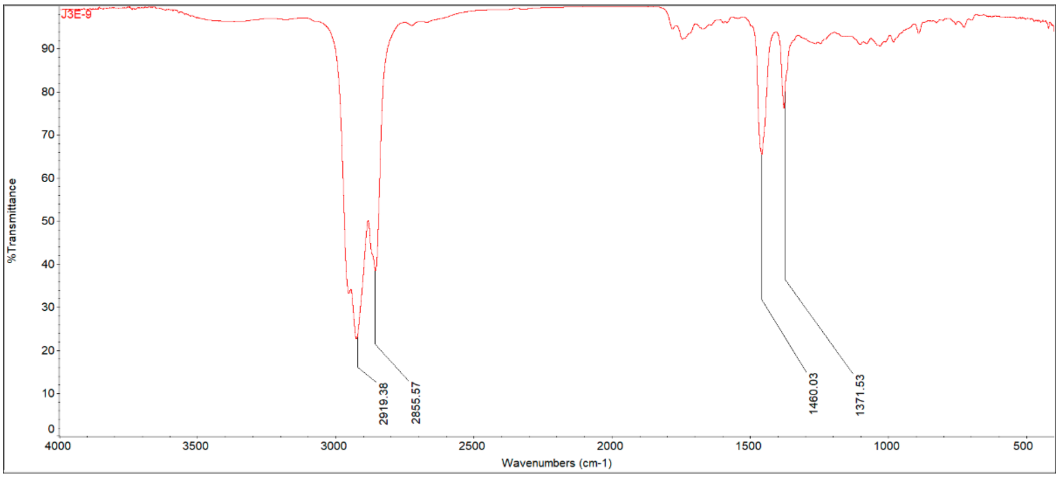

Figure S8. IR spectrum of compound **1**.

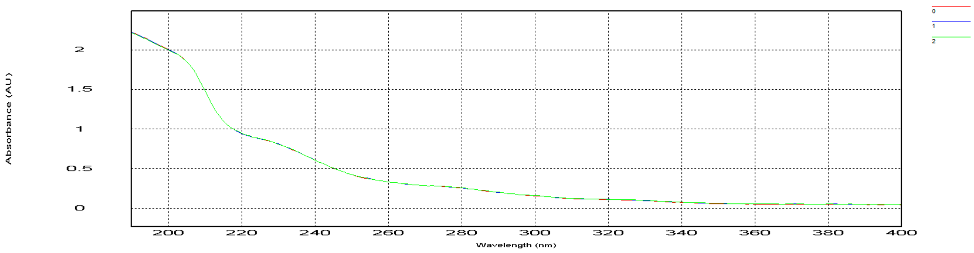

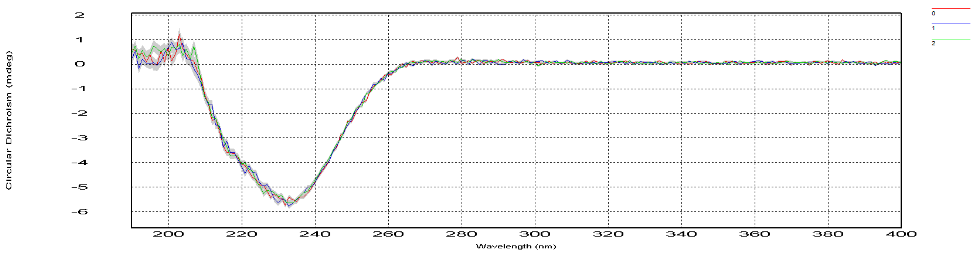

Figure S9. UV and CD spectrum of compound **1.**

2. Spectra of compound **2**

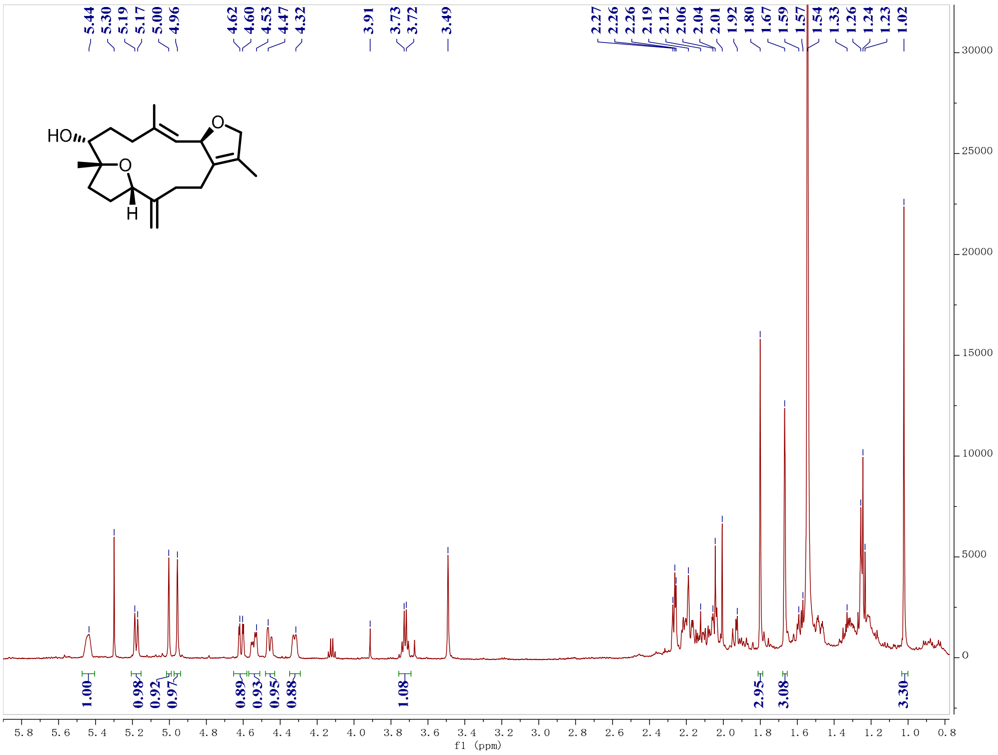

Figure S10. ^{1}H NMR spectrum (600 MHz) of compound **2** in $CDCl_3$.

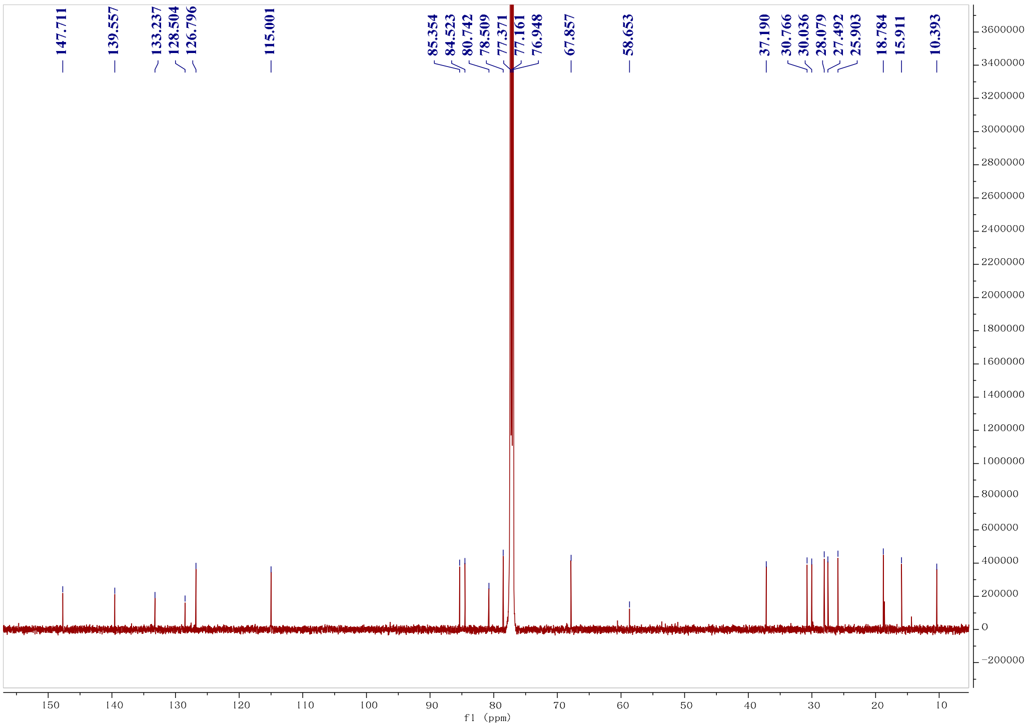

Figure S11. ^{13}C NMR spectrum (600 MHz) of compound **2** in $CDCl_3$.

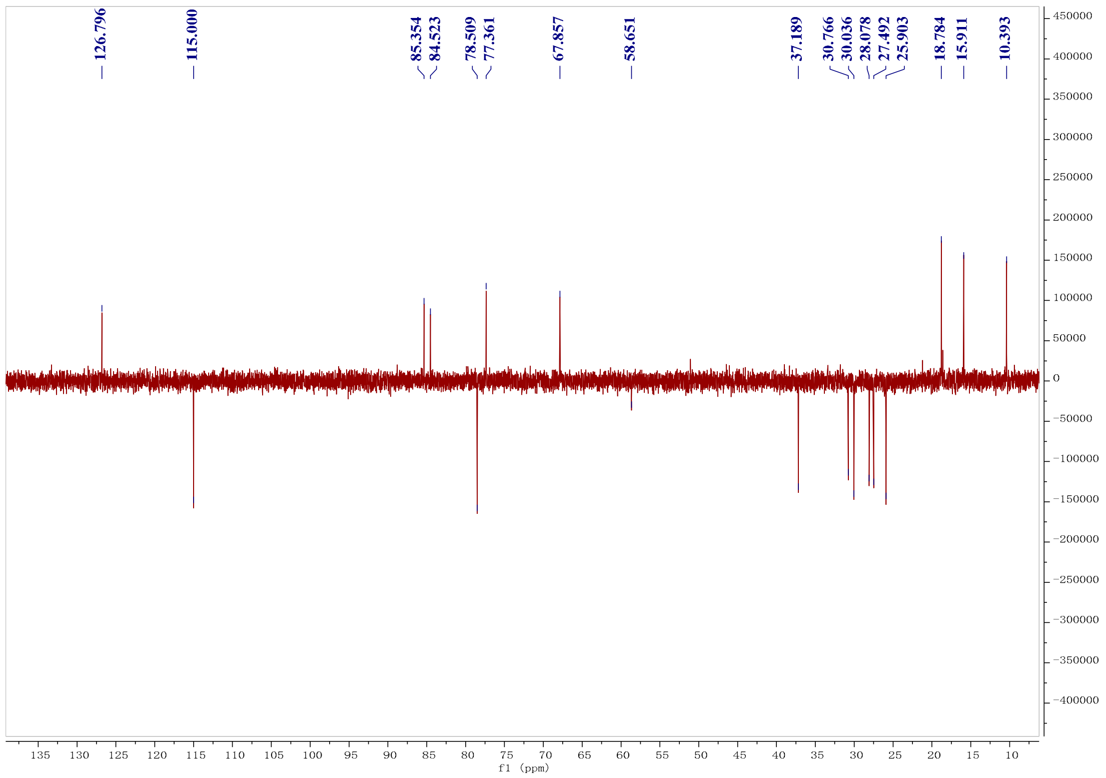

Figure S12. DEPT spectrum (150 MHz) of compound **2** in $CDCl_3$.

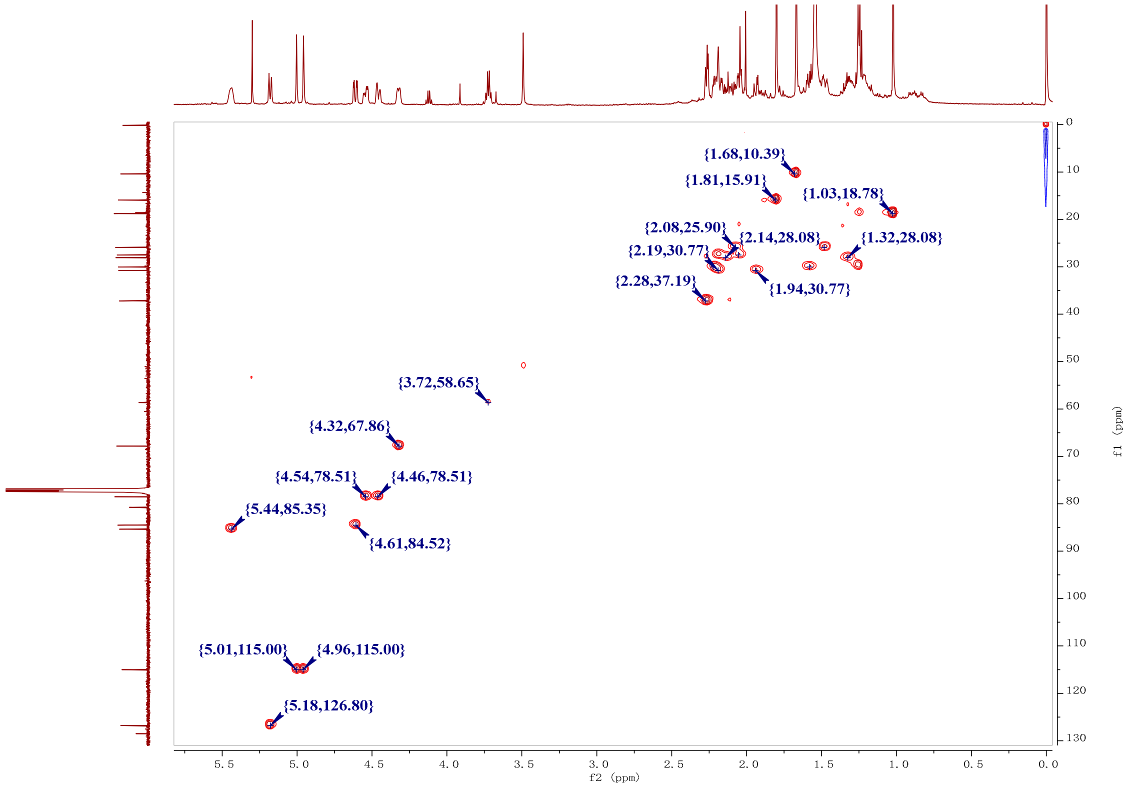

Figure S13. HSQC spectrum (600 MHz) of compound **2** in $CDCl_3$.

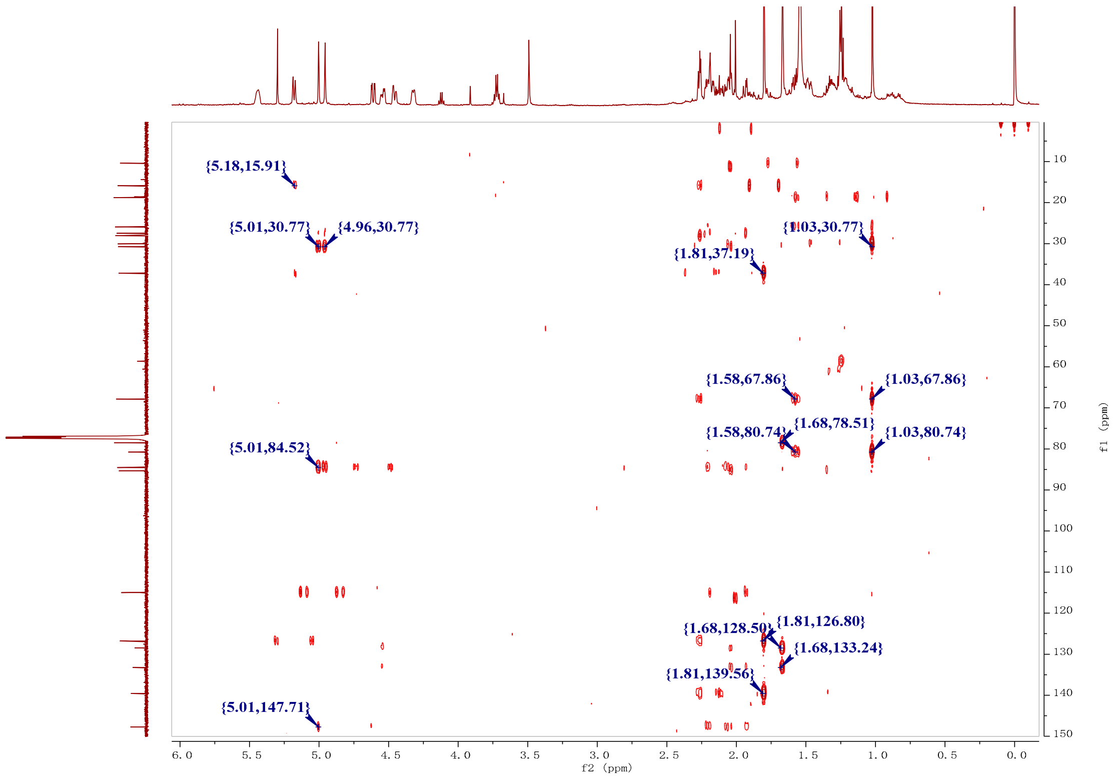

Figure S14. HMBC spectrum (600 MHz) of compound **2** in $CDCl_3$.

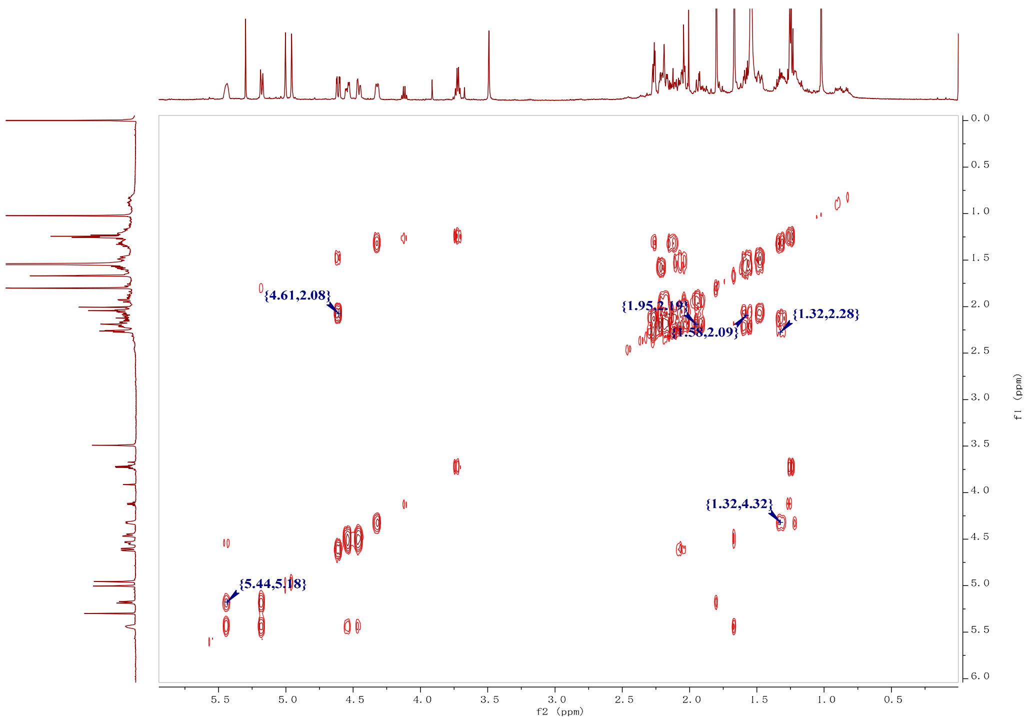

Figure S15. ^1H–^1H COSY spectrum (600 MHz) of compound **2** in $CDCl_3$.

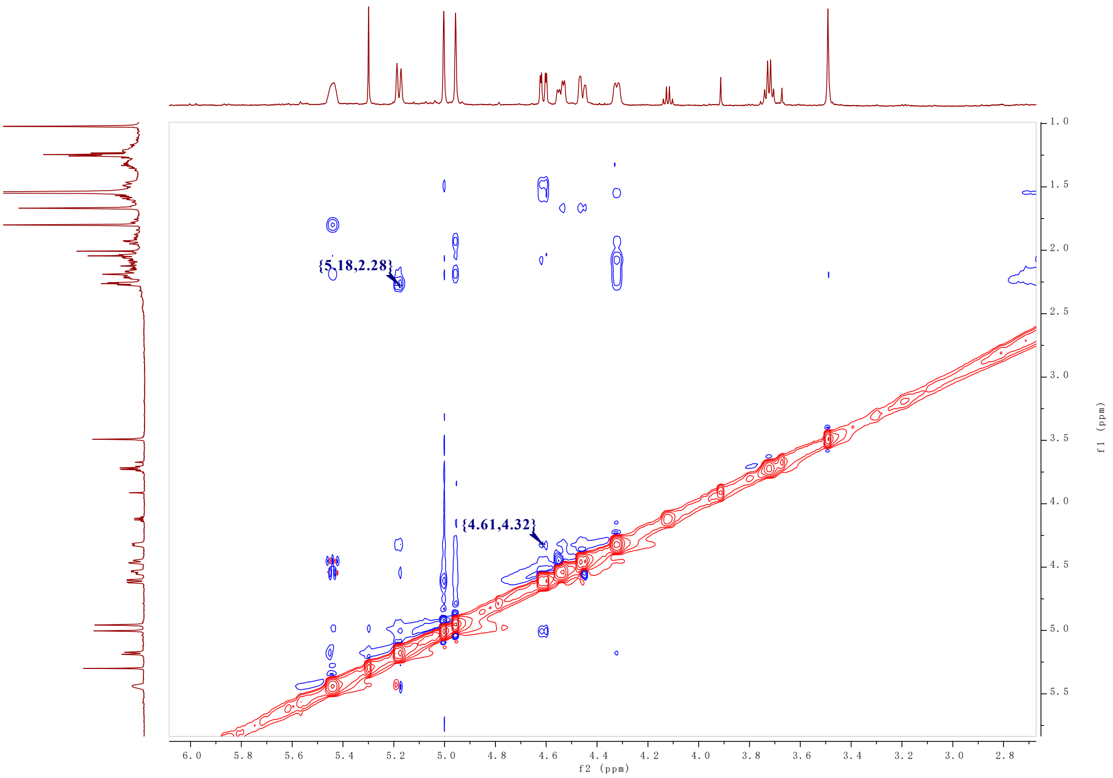

Figure S16. NOESY spectrum (600 MHz) of compound **2** in $CDCl_3$.

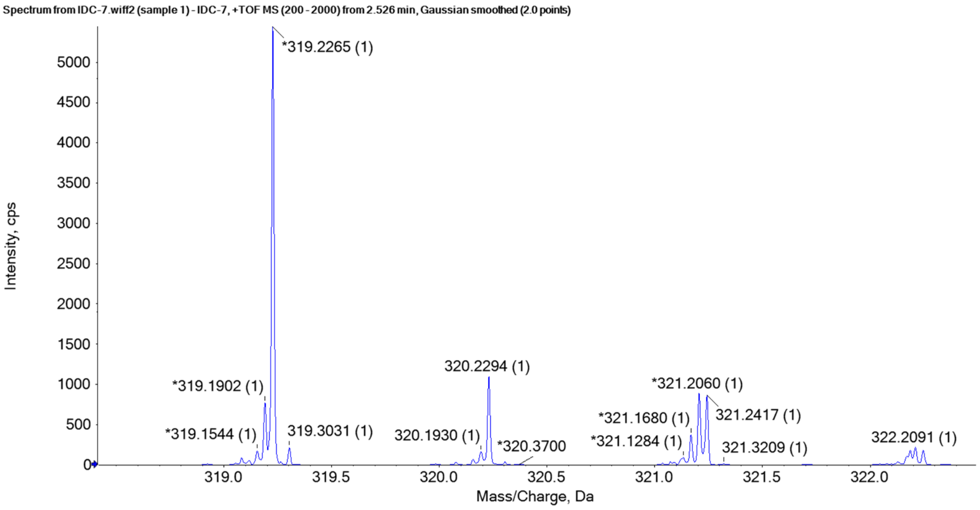

m/z	Cal m/z	Error(mmu)	Error(ppm)	Ion Formula	Ion
319.2265	319.2268	-0.3	-0.8	$C_{20}H_{31}O_3$	$[M+H]^+$

Figure S17. HR-ESI-MS spectrum of compound **2**.

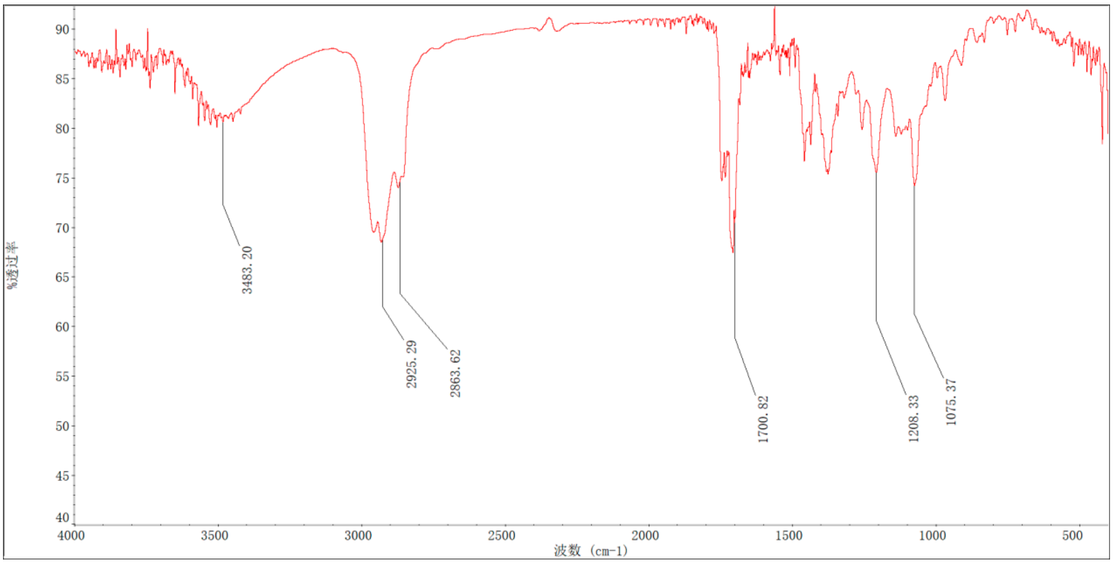

Figure S18. IR spectrum of compound **2**.

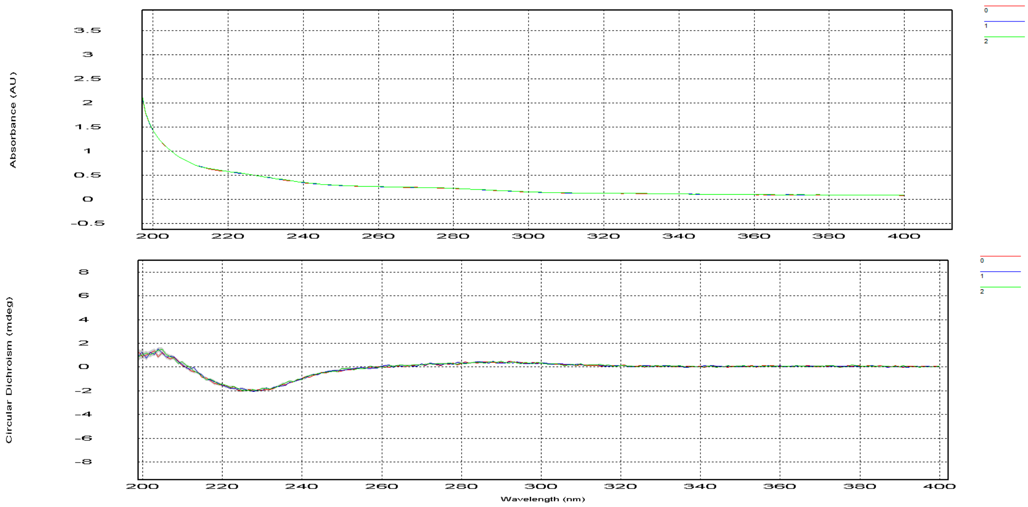

Figure S19. UV and CD spectrum of compound **2**.

3. Ouantum chemical calculations of NMR shifts for compound **2**.

3.1 Structures of isomers studied for compound **2**.

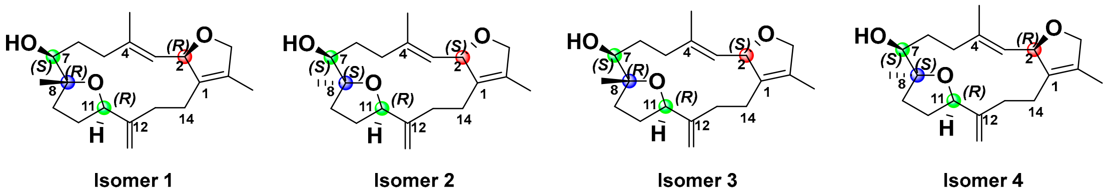

Figure S20. Structures of isomers of compound **2**.

3.2. Isotropic magnetic shielding constants of compound **2**.

Table S2. Boltzmann averaged GIAO isotropic magnetic shielding constants (σ) of compound 2 calculated at the PCM/mPW1PW91/6-31G*//B3LYP/6-311G(d,p) level of theory.

	Isotropic shielding constants			
Nuclei	2a	2b	2c	2d
C 1	46.0	46.8	44.3	43.7
C 2	97.4	97.9	93.8	97.0
C 3	52.4	50.8	55.5	53.0
C 4	38.3	38.0	39.2	36.6
C 5	147.4	147.5	148.5	149.6
C 6	152.4	154.5	153.5	156.0
C 7	112.3	105.7	110.0	107.7
C 8	99.7	96.2	99.2	97.6
C 9	145.9	146.7	145.8	146.8
C10	149.4	153.2	151.5	151.8
C11	106.2	98.0	103.2	102.0
C12	27.59	28.31	23.89	28.43
C13	147.90	157.74	149.85	145.96
C14	161.45	161.84	155.66	160.81
C15	47.39	47.59	47.68	49.31
C16	175.10	175.25	175.30	175.10
C17	105.18	105.84	105.61	105.55
C18	171.61	170.83	168.23	169.62
C19	166.72	162.32	166.41	162.20
C20	71.35	68.95	71.20	66.25
H 2	26.24	26.19	26.17	26.22
H 3	26.25	26.15	26.03	26.27
H 5	29.61	29.05	29.18	29.55
H 5	28.93	29.66	29.62	29.36
H 6	29.94	29.65	29.72	30.02
H 6	29.85	29.96	30.09	29.72

H 7	28.23	28.22	28.19	28.23
H 9	29.99	30.10	29.82	29.61
H 9	29.68	29.56	29.96	30.09
H10	29.76	29.88	30.30	30.08
H10	30.46	30.00	29.83	29.79
H11	27.60	27.27	27.53	27.49
H13	29.36	29.05	29.57	29.30
H13	29.52	29.66	29.20	29.49
H14	29.75	28.89	29.69	29.28
H14	29.37	29.59	29.03	29.51
H16	30.08	30.20	30.28	30.09
H16	29.66	29.58	29.70	29.68
H16	30.26	30.16	30.29	30.27
H17	27.01	27.26	27.21	27.03
H17	27.20	27.05	27.03	27.15
H18	29.42	29.39	29.05	29.55
H18	30.17	30.33	29.80	29.95
H18	30.31	29.72	30.25	30.35
H19	30.41	30.54	30.45	30.56
H19	30.40	30.44	30.82	30.48
H19	30.72	30.83	30.47	30.81
H20	26.65	26.63	26.45	26.41
H20	26.15	26.32	26.27	26.28

3.3. DP4+ results of compound **2**.

Table S3. DP4+ results obtained using experimental data of compound **2** versus isomers 2a, 2b, 2c and 2d.

Functional		Solvent?		Basis Set		Type of Data	
mPW1PW91		PCM		6-311+G(d,p)		Shielding Tensors	
	Isomer 1	Isomer 2	Isomer 3	Isomer 4	Isomer 5	Isomer 6	
sDP4+ (H data)	0.02%	99.97%	0.00%	0.02%	–	–	
sDP4+ (C data)	0.19%	32.12%	47.54%	20.15%	–	–	
sDP4+ (all data)	0.00%	99.99%	0.00%	0.01%	–	–	
uDP4+ (H data)	1.13%	98.50%	0.02%	0.35%	–	–	
uDP4+ (C data)	0.36%	88.31%	10.57%	0.76%	–	–	
uDP4+ (all data)	0.00%	99.99%	0.00%	0.00%	–	–	
DP4+ (H data)	0.00%	100.00%	0.00%	0.00%	–	–	
DP4+ (C data)	0.00%	84.56%	14.99%	0.46%	–	–	
DP4+ (all data)	0.00%	100.00%	0.00%	0.00%	–	–	
